# Mapping global development potential for renewable energy, fossil fuels, mining and agriculture sectors

**DOI:** 10.1038/s41597-019-0084-8

**Published:** 2019-06-27

**Authors:** James R. Oakleaf, Christina M. Kennedy, Sharon Baruch-Mordo, James S. Gerber, Paul C. West, Justin A. Johnson, Joseph Kiesecker

**Affiliations:** 1 Global Lands Program, The Nature Conservancy, Fort Collins, CO 80524 USA; 2 Global Landscapes Initiative, Institute on the Environment, University of Minnesota, St. Paul, MN 55108 USA; 3 Natural Capital Project, Institute on the Environment, University of Minnesota, St. Paul, MN 55108 USA

**Keywords:** Environmental impact, Environmental impact, Socioeconomic scenarios

## Abstract

Mapping suitable land for development is essential to land use planning efforts that aim to model, anticipate, and manage trade-offs between economic development and the environment. Previous land suitability assessments have generally focused on a few development sectors or lack consistent methodologies, thereby limiting our ability to plan for cumulative development pressures across geographic regions. Here, we generated 1-km spatially-explicit global land suitability maps, referred to as “development potential indices” (DPIs), for 13 sectors related to renewable energy (concentrated solar power, photovoltaic solar, wind, hydropower), fossil fuels (coal, conventional and unconventional oil and gas), mining (metallic, non-metallic), and agriculture (crop, biofuels expansion). To do so, we applied spatial multi-criteria decision analysis techniques that accounted for both resource potential and development feasibility. For each DPI, we examined both uncertainty and sensitivity, and spatially validated the map using locations of planned development. We illustrate how these DPIs can be used to elucidate potential individual sector expansion and cumulative development patterns.

## Abstract (summary)

**Table:** 

Design Type(s)	data integration objective • modeling and simulation objective • population modeling objective
Measurement Type(s)	land conversion process
Technology Type(s)	digital curation
Factor Type(s)	sector • geographic location • material_entity
Sample Characteristic(s)	Earth (Planet) • anthropogenic habitat • fossil fuel • cropland ecosystem • natural environment • mineral deposit

Machine-accessible metadata file describing the reported data (ISA-Tab format)

## Background & Summary

Human activities have transformed most of the world’s terrestrial landscapes^1^, resulting in accelerated resource exploitation, environmental deterioration, biodiversity loss, and climate change^2–4^. Growing human populations^5^ and increasing wealth in many regions^6^ will inevitably propel further development to meet the rising demands for food, water, energy, and other land-based resources. Predicting and managing forthcoming development is essential to minimize the impacts of large-scale expansion on natural habitats and their services and to promote ecological and socioeconomic sustainability^7^. Anticipating where future development may occur requires mapping of land that is suitable to human activities^8^ (e.g., growing crops, expanding housing development, establishing a mine), often taking into consideration the area’s biophysical criteria (e.g., prevailing climate, soil, topography), land use or administrative constraints (e.g., compatible land types, protected areas), and/or socio-economic factors (e.g., accessibility to markets or infrastructure) that are associated with the target development^9,10^.

Global land suitability mapping has aided our understanding of the expansion of many development sectors, including cropland or biofuel expansion, renewable energy sources (i.e., solar, wind, and hydropower), fossil fuels, and mining. However, previous global assessments for food and biofuel suitability are largely binary maps for “croplands” (e.g.^11–13^) or focus on marginal and abandoned land potential for biofuel production (e.g.^14,15^). A few cropland assessments account for social or policy constraints (e.g.^13,16^), but none globally map feasibility of land conversion based on factors of yield potential and access to infrastructure to distinguish relative conversion pressure^8^. Global mapping of renewable energy potential maps have incorporated only simple land constraints^17–19^ or select few spatial development feasibility factors (e.g., market accessibility that considers distance to urban areas, load centers, and transmission lines^20–24^, or site construction and operational costs^21,24,25^), at times doing so only post-hoc to categorize potential energy production^26,27^ or to compare implementation costs^23,24^. Global fossil fuels and mining sectors maps have been limited to one fuel or mineral type^28–31^, do not include spatial siting factors^20,32^, or rely on proprietary industry data that limits public distribution^33^. The inconsistency in mapping across different sectors and the lack of publicly available maps at resolutions finer than large-scale aggregate summaries (e.g., countries) severely limits the ability to plan for cumulative development pressures across geographic regions.

Here, we generated spatially-explicit, global land suitability maps at a fine resolution (1-km) for renewable energy (concentrated solar power – CSP, photovoltaic solar power – PV, wind power – Wind, and hydropower – Hydro); fossil fuels (coal mining – Coal, conventional oil – CO, conventional gas – CG, unconventional oil – UO, and unconventional gas – CG); mining (metallic minerals – MM and non-metallic minerals – NMM); and agriculture (crop expansion – Crop and biofuels expansion – Bio) development using publicly available datasets that account for both resource potential and development feasibility^34^. For each of the 13 sectors, we produced a land suitability index, referred to as a “development potential index” (DPI), that relatively ranks each 1-km area of land for its likelihood to be modified in the future by that sector and then classified each sector consistently on a high-low scale based on its DPI values. The sector-specific DPI datasets are made freely and publicly available to the scientific community and to policy decision-makers to facilitate broad-scale spatial assessments of potential individual sector and cumulative development patterns, and can be used to identify high-risk areas where near-future expansion may conflict with biodiversity, climate, or environmental assets.

**Fig. 1:**
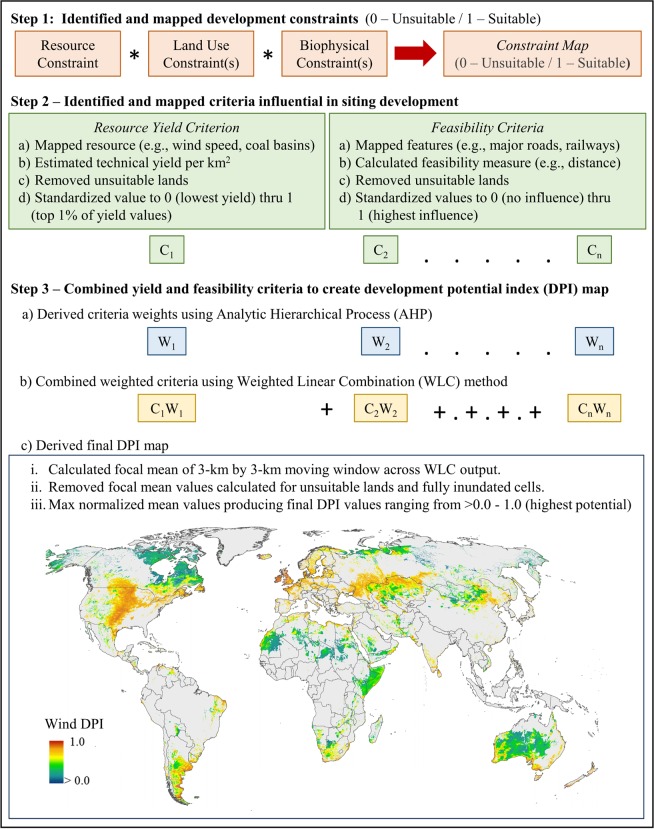
Procedures used to produce all development potential index (DPI) maps. Analysis steps were applied for the 13 sectors related to renewable energy, fossil fuels, mining, and agriculture.

We created DPIs using three mains steps of spatial multi-criteria decision analysis (MCDA) techniques in geographic information systems (GIS) (Fig. 1)^9^. First, we mapped sector-specific land constraints expected to restrict development (e.g., suitable land cover, slope). Second, we produced spatially-explicit, independent criteria that were continuously-scaled factors that enhanced the suitability of sector development^35^, and captured both resource availability (sector-specific yields) and development feasibility (e.g., distance to major roads, railroads, ports, power plants, electrical grid, and demand centers). We removed areas with constraints from the mapped resource yield and development feasibility criteria and standardized values from 0–1. Third, we weighted the importance of spatial criteria using Analytic Hierarchy Process (AHP)^36–38^, and combined them into DPI maps using Weighted Linear Combination (WLC) in GIS^9^. We analyzed both output uncertainty and input sensitivity following refs^39,40^ and validated our DPIs using over 6,000 points and 200,000 km^2^ of mapped locations identifying recent or planned development sectors. While GIS within MCDA procedures (GIS-MCDA), specifically AHP in combination with WLC, have been widely used to map land suitability at local and regional scales^10,41^ (e.g., siting of renewable energy facilities^42–46^, fossil fuel development^47–49^, mineral extraction^50^, and agriculture development^51–53^), to our knowledge, this is the first study to apply these procedures consistently across multiple energy, extractive, and agricultural sectors on a global scale.

## Methods

To inform parameters and criteria selection for each step of the analysis, we conducted a literature review of studies on the mapping of land suitability, yield potential, and/or economic feasibility associated with all development sectors (Online-only Table 1). Because technologies in all development sectors are rapidly changing, we limited our literature search to papers published within the last 10 years focusing mainly on global^17–25,27,32,54^ and regional^16,55–60^ analyses but also using state/local analyses^44–51,53,61–66^ to fully capture the variety and weights of criteria used in all analyses. We relied on the most commonly cited development constraints and criteria that could be mapped from publicly available, open access global data to produce our DPI maps and thus facilitate public distribution of derived datasets.

Analyses were performed at a 1-km resolution for terrestrial lands defined as cells containing one or more 300-m pixels of terrestrial land cover types based on ESA CCI dataset^67^. We projected input data to the equal-area Mollweide projection and applied bilinear resampling method for continuous raster data and nearest-neighbor method for discrete data (vector data were first projected and then converted to raster datasets). Unless otherwise specified, analyses were conducted using ArcGIS 10.5 (www.esri.com) with the Spatial Analyst 10.5 extension.

### Mapping development constraints (step 1)

Constraints were tied to resource thresholds (e.g., solar irradiance, wind speed), land use characteristics (e.g., urban areas), and biophysical characteristics (e.g., slope, elevation) that limit the ability of the sector to economically produce its associated commodity or to be constructed. Addition of these spatial constraints improved each sectors’ DPI by limiting the extent of the analysis to only viable locations^16^. Given the wide-range of constraints and their values often reported in the literature, we selected the least-restrictive constraint values reported by global or regional studies (see Online-only Table 1) to avoid the exclusion of areas that may become accessible to future development with improved technology. We did not include any administrative constraints (e.g., excluding protected areas) because these assignments can be modified or removed based on policy changes and land use pressure^68^. Online-only Table 2 provides sector-specific breakdown of the constraints applied with appropriate citations with their justifications, which we summarize briefly below.

For solar and wind renewable energy sectors (CSP, PV, Wind), we excluded areas below sector-specific resource (solar irradiance or wind speed) and above slope or elevation thresholds, and areas categorized as snow, ice, or urban. For CSP, we also excluded areas with already operating CSP power plants, and for wind, we excluded areas with ≥3 wind turbines per km^2^ (we did not exclude existing PV power plants given lack of global data). For Hydro, we excluded urban areas, and locations with existing hydroelectric dams or estimated to produce <1 MW of power.

We excluded lands classified as urban for all fossil fuel and mining sectors. For coal mining, we also excluded existing coal mines based on our mapping from global sources, and for mineral and non-mineral mining, we excluded lands with former or current active mines. We did not exclude current oil and gas wells due to the lack of publicly available, globally comprehensive data on well locations. Lastly, for agriculture sectors, we removed lands classified as urban or currently cropped, arid lands without irrigation, and areas too steep for cultivation.

### Mapping development criteria (step 2)

#### Mapping resource yield criteria

For each sector, we spatially mapped a resource yield criterion based on available resources converted to yield estimates using common production values (e.g., annual megawatt hours, barrels of oil, tons of coal) for each 1-km^2^ cell. See Online-only Table 2 for detailed methods and data applied to derive these resource yield maps with brief descriptions below. We limited the resultant global maps to suitable locations based our constraint maps (step 1), and applied the following steps on the yield map values to produce approximately normally distributed values ranging from 0–1 across all sectors: (1) reassigned values of cells that were within the top one percentile outliers to the 99^th^ percentile value of the distribution, (2) applied transformation based on the skewness (*s*) of the yield value distribution as follows: no transformation if *s* < 0.5, square-root transformation for 0.5 ≤ *s* ≤ 1.0, and log-transformation if *s* > 1^69,70^, and (3) scaled data into a 0–1 range using min-max normalization. This approach addressed the right skewed distribution of most yield cell values and maintained all cells but treated the top 1% of outlying yield values as a constant value given their expected lack of differentiation in development potential.

Renewable energy: We estimated yield for four renewable energy sectors: CSP, PV, Wind, and Hydro (Online-only Table 2). For solar (CSP and PV) and wind, we used the general equation: *PD ∙ CF*_*i*_ ∙ 8760 to estimate annual yield (MWh/km^2^), where *PD* is the sector-specific power density in MW/km^2^, *CF*_*i*_ is the sector-specific and spatially explicit (for the *i*th cell) capacity factor derived from the corresponding resource estimate (e.g., wind speed for wind) and defined as the ratio of expected to potential power output, and 8760 is the number of hours in a year^26,57,59^. For wind, we multiplied this equation by a spatially-explicit air density factor (*AD*_*i*_), because differences in elevation have known effects on wind power production^17,27^. For hydropower, we used a publicly available, 1-km resolution hydropower potential dataset which derived potential from a global digital elevation model and river runoff data using a fixed *CF* value of 0.5^18^.

Fossil fuels: We estimated yield for five fossil fuel energy sectors: Coal, CO, CG, UO, and UG (Online-only Table 2). Yields were derived from global and national level assessments of technically recoverable resources per basin (coal), assessment units (CO, CG, UO, and UG), or prospective areas (UO and UG), which are collectively referred to as assessment units (AUs). For each AU, we divided the total recoverable resource by the AU area, thereby producing an average yield/km^2^ across the AU. Where AUs overlapped, we summed resource-specific (e.g., conventional oil) yield values before producing the final yield map.

Mining: Due to the variety of different minerals mined and the lack of global, basin-level estimates of technically recovered minerals for each, we relied on proxy yield values based on deposit locations for two collective categories of mining: metallic and non-metallic (Online-only Table 2). Mineral deposit data were publicly available for 167 minerals along with categorical size estimates of deposit amounts that were determined from attribute size descriptions (e.g., very large, large, medium, etc.). Given the availability of only categorical estimates and because deposit densities are widely used to estimate undiscovered deposits and potential recoverable amounts^71,72^, we relied on mining density as a surrogate for yield. We implemented kernel density (KD) methods^73^ using Kernel Density tool in ArcGIS, and incorporated categorical deposit amounts as weights following ref.^74^. Kernels were centered on each deposit location and generated based on deposit size weights and on radii distances specific to metallic or non-metallic minerals. We then selected only cells with KD > 0.001 deposits/km^2^, a minimum value used by ref.^75^ and https://www.openstreetmap.org/. Because 82% of mineral deposits were located within the U.S., we created and standardized KD maps separately for the U.S. and non-U.S. regions before combining these two regions to produce each sector’s final resource yield map.

Agriculture: We estimated agriculture yield for ten food crops, which captured 83% of total calorie production on croplands^76^, and for a subset of five first-generation biofuel crops, which comprised the majority of commercial biofuel production^77^ and have the most growth potential based on market maturity and technological capacity^78^ (Online-only Table 2). Using 2012 yield data summarized at national or sub-national jurisdictional units, and following methods in ref.^79^, we modeled the crop-specific relationships between area-weighted yield (ton/km^2^) and biophysical covariates (e.g., growing degree-day, precipitation, fraction irrigated, slope, etc.) using a 95th percentile quantile regression to predict attainable yields (quantreg package version 5.33 in R version 3.4.0; model coefficient results in Online-only Table 3). We then combined spatially-explicit covariate maps with the resulting model coefficients to produce global, crop-specific, predicted yield maps. For crop expansion, we min-max normalized these yield values for each crop. For biofuel crops, we converted predicted yield in ton/km^2^ to gallons of gasoline equivalents (GGE) per km^2^ based on conversion rates from ref.^20^. We generated final cropland and biofuel resource potential maps by calculating the mean standardized yield value (i.e., 0–1 for crops and GGE/km^2^ for biofuels) across the ten subsistence crops and five biofuel crops respectively, and then ensured normal distribution of these two final yield maps following the methods discussed previously.

#### Mapping feasibility criteria

We produced 13 development feasibility criteria at 1-km resolution, which were factors that increase site development potential or decrease operational costs (details in Online-only Table 4). These criteria related to: (1) ability to transport resources and/or construction materials (distance to major roads, railways, and ports); (2) access to resource demand centers (market accessibility, distance to the electrical grid, urban areas, coal-fired power plants, and aggregate demand centers); (3) locations of existing development (distance to producing oil and gas fields and active coal mining density); and (4) other economic costs associated with resource siting (inverse population density), development (landcover feasibility and land supply elasticity), and/or production (access to electricity). Criteria values ranged from 0 to 1, with 1 indicating the most preferred location for a particular sector development associated with the criteria, and 0 implying the criteria no longer provided any advantage for this development. For each sectors’ MCDA, we selected criteria used in previous studies on land suitability or recognized as an economic factor influencing siting (Online-only Table 1), and that could be mapped globally from existing, publicly available data.

We generated distance criteria values (*c*) based on a Gaussian distance decay function, *c* = exp(−*d*^2^/2∙(*h*/2)^2^), where *d* is Euclidian distance between a focal cell and the closest feature of influence (e.g., transmission line, roads, railway, etc.), and *h*/2 is the inflection point, or the point beyond which the criteria score starts to rapidly decline towards zero. Resulting standard values of *c* ranged from 0–1, where 1 indicated closest proximity to the feature of importance. For renewable energy sectors, we set *h* to 100 km similar to ref.^56^, and for fossil fuels and mining sectors, we set *h* to 50 km, or the average distance at which infrastructure costs associated with mineral extraction approximately doubles^80^.

### Combining yield and feasibility criteria to create DPIs (step 3)

We used Analytic Hierarchy Process (AHP)^36,81^ and Weighted Linear Combination (WLC)^9^ methods to combine resource yield and feasibility criteria maps into a final DPI map for each sector. AHP calculates criteria weights from a pairwise matrix of importance values (judgement matrix) formulated based on scaled rankings^36,37^. Pairwise comparison values are assigned based on Saaty’s nine-point importance scale^36,37^, where a score of 9 indicates criterion A is nine times more important than criterion B, and where scores are reciprocal (therefore criterion B is 1/9 times as important as criterion A). We selected criteria, none of which were highly correlated (Pearson’s correlation > 0.6) and assigned importance values based on our literature review of sector suitability (Online-only Table 1), current literature on resource transportation and development costs^80,82–113^, and authors’ expertise. Criteria weights were calculated as the normalized eigenvector associated with the judgement matrix’s largest eigenvalue (*λ*_*max*_) (see refs^36,37^ for details). We evaluated the consistency of the judgement matrices using a consistency ratio (*CR*) calculated as *CR* = *CI*/*RI*, where *CI* is the consistency index calculated based on number of criteria (*n*) as *CI* = (*λ*_*max*_ − *n*)/(*n* − 1), and *RI* is an established random inconsistency index based on *n* (see Table 1.2 in ref.^81^).

We found all judgment matrices to be within acceptable consistency (i.e., CRs < 0.10). For all judgement matrices, the resource yield criterion was identified as the most important, which produced weight ranging from 0.336 (coal) to 0.552 (metallic mining). Judgement matrices of fossil fuel sectors included feasibility criteria related to current development, which were prioritized as next highest, or equal to, resource yield criteria. For all other sectors, the typical second highest prioritized criteria were related to transporting the resource to its respective demand centers, except for hydropower, for which population avoidance (i.e., inverse population density criterion) ranked as second highest. Supplementary Table S1 provides judgement matrices for each sector including justifications for importance ranking, and derived weights used to calculate DPIs.

Once we derived the resource yield and feasibility criteria maps with their associated weights, we implemented WLC in ArcGIS using the Weighted Overlay tool to create sector-specific DPI maps using: ∑*w*_*n*_*c*_*n*_, which produces a composite DPI map based on AHP derived weights (*w*_*n*_) for *n* criteria (*c*_*n*_)^9^. Values of all input criteria maps and resulting DPI maps ranged from >0 (low) to 1 (high). To reduce local variations as a result of global data inaccuracies or resolution artifacts, we spatially averaged DPI values using a 3 × 3-km moving window analysis^10^. If any cell previously excluded based on constraints or water was assigned a value by the smoothing technique, we reassigned its value to null. A final continuous DPI was created by max normalizing the remaining spatially-averaged values^34^ (e.g., Fig. 1-Step 3iii).

#### DPI classification

To facilitate the comparison of spatial patterns across the different sectors, we grouped the continuous DPI values into six relativized development potential classes: very high, high, medium high, medium low, low and very low. Given that each sector’s DPI values were approximately normally distributed but varied in their mean values, we calculated the standard global z-score per pixel and then binned each DPI based on five z-score breakpoints that corresponded to the percentiles of the distribution (Table 1). This method classified each DPI equally based on its mean and standard deviation of values using consistent estimated percentage breakpoints of 10% (Very Low, Very High), 15% (Low, High), and 25% (Medium-high, Medium-low). These breakpoints are offered as one way to classify the continuous DPI values and are included in each sector-level DPI data bundle^34^ (e.g., Supplementary Table S1). We note that these six classes have been applied in previous global threat analyses (e.g., by ref.^114^ when classifying cumulative threats to global marine environments).

**Table 1:** Development potential index (DPI) classes.

DPI Class	Standard z-score range*	Estimated percentile range*
Very High	>1.282	>90^th^ percentile
High	0.675–1.282	75^th^ percentile–90^th^ percentile
Medium-high	0.000–0.675	50^th^ percentile–75^th^ percentile
Medium-low	−0.675–0.000	25^th^ percentile–50^th^ percentile
Low	−1.282–−0.675	10^th^ percentile–25^th^ percentile
Very Low	<=−1.282	<=10^th^ percentile

Standard z-score ranges used to define Development Potential Index (DPI) classes and estimated percentile data ranges based on normally distributed values.

*Highest value in range included in class (e.g. z-score 1.282 is assigned to High DPI class).

### Uncertainty and sensitivity analyses

For each DPI sector, we quantified the variability associated with each output based on model input (i.e., uncertainty analysis), and then identified which DPI criteria were responsible for the most variability (i.e., sensitivity analysis).

#### Uncertainty analysis

The two main sources of uncertainty for any MCDA arise from the criteria maps and their weights^9,115^. For the criteria maps, uncertainty can stem from three aspects in the analysis; (1) choice of criteria in the decision model, (2) errors of measurement in the original source spatial data, and (3) the value scaling (or standardization) of the criterion maps^115^. To reduce these sources of uncertainty, we respectively (1) relied on supported literature to guide criteria selection and value scaling of categorical data (Online-only Table 1), (2) avoided arbitrary classifications of continuous input data, and (3) applied fuzzy measures when appropriate. We focused our uncertainty analysis on the weighting values, a common approach when addressing uncertainty regarding GIS-MCDA^39,116–118^. To do so, we relied on a Monte Carlo (MC) approach, which assesses both qualitative and quantitative uncertainty within an MCDA based on repeated random sampling from a range of criteria weights to produce several iterations of the model results^118^. These iterations are then used to calculate standard deviation (SD) and/or coefficient of variation (CV) to map and analyze uncertainty^39,116,117,119^.

To define our weight ranges for each DPI criterion, we modified the original AHP by first increasing the importance value of a selected criterion by two points across all other criteria and then used this new matrix to calculate the maximum weight for this criterion weight range. We then decreased the importance value for this same selected criterion by two points across all criteria to derive the minimum weight for the range. This process emulated selecting the next highest or lowest odd number comparison value (i.e., 1, 3, 5, 7, 9 values most commonly used when assigning values using the Saaty’s nine-point importance scale^36^), while also maintaining the necessary consistency ratio (i.e., CR < 0.1) to use these derived weights (see the Supplementary Information for an example). Once the weight ranges for all criteria were defined (Online-only Table 5), we followed standard MC methodology and applied a four step process similar to refs^39,120^ for each DPI. For 300 iterations, we: (1) randomly selected a criterion, (2) randomly assigned the criterion weight from its bracketed range of generated weights (Online-only Table 5), (3) proportionally modified the remaining criteria weights such that all weights sum to the value of one, and (4) applied these weights to reproduce the modified DPI. Our chosen number of 300 iterations falls within the recommended range of 100–10000^121,122^ and predominantly produced a <1% change of the mean and SD associated with each iteration as the iterations neared 300 which suggested this number was sufficient^117^.

Based on mean CV across all DPI maps, the biofuel and crop DPIs exhibited the highest relative uncertainty, largely attributed to these DPIs having the lowest number of criteria (Table 2). In contrast, the wind DPI had the lowest uncertainty, likely driven by this sector having the greatest number of criteria. This inverse relationship between uncertainty and the number of criteria was upheld by all sectors except coal and unconventional oil (Table 2). Coal had higher than expected uncertainty presumably because of the larger undeveloped basins located in remote regions of the globe. Unconventional oil basins on the other hand are found in more accessible locations and consistently much smaller in size in comparison to other fossil fuel sectors; thus, limiting the variability caused by development feasibility criteria and thus also reducing uncertainty.

**Table 2:** Mean coefficient value (CV) for 13 development sectors with number of criteria used for each development potential index (DPI).

DPI Sector	Mean CV	Number of Criteria in MCDA
Biofuels Expansion	0.1373	3
Crops Expansion	0.1076	3
Metallic Mining	0.0798	4
Non-metallic Mining	0.0701	4
Coal Mining	0.0664	6
Conventional Gas	0.0609	5
Unconventional Gas	0.0572	5
Conventional Oil	0.0527	5
Photovoltaic Solar Power (PV)	0.0480	6
Hydropower	0.0450	6
Concentrated Solar Power (CSP)	0.0439	6
Unconventional Oil	0.0415	5
Wind Power	0.0279	7

Sectors ordered from largest to smallest mean CV.

**Fig. 2:**
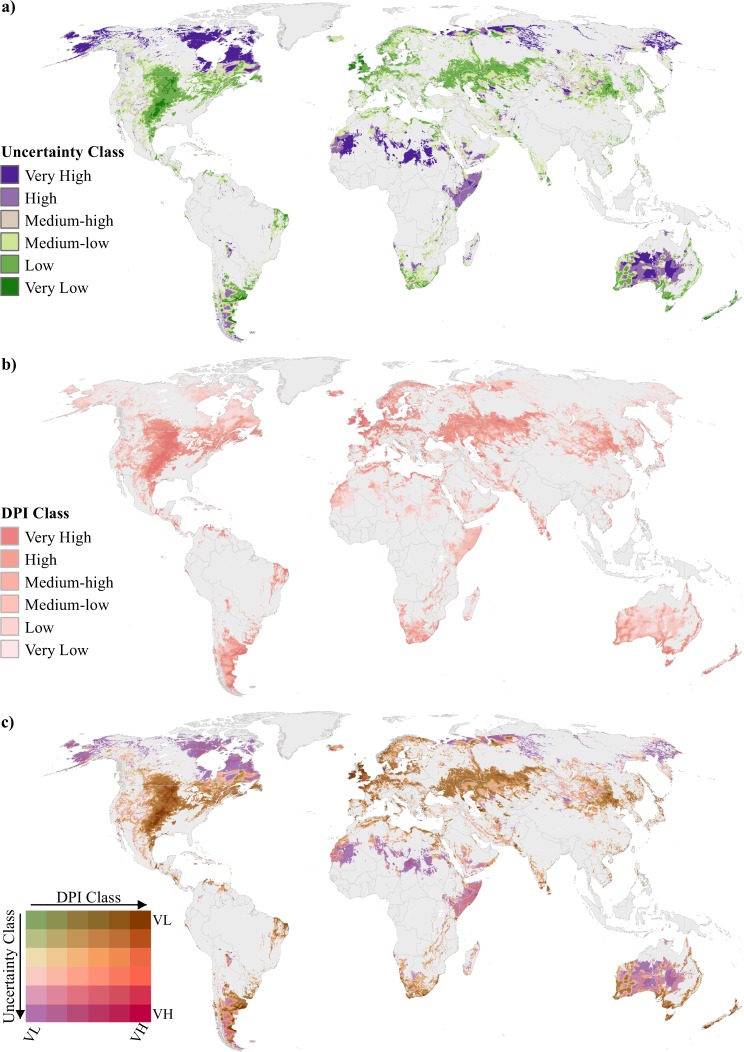
Example spatial uncertainty analysis for wind development potential index (DPI). Spatial datasets used for wind DPI uncertainty analyses: (**a**) classified wind uncertainty map, (**b**) classified wind DPI map, and (**c**) resulting map produced by intersection of two maps. In the legend for map (**c**), arrows indicate the direction of classes going from “Very Low” (VL) to “Very High” (VH). For example, purple areas classified as VL for DPI and VH for uncertainty, whereas dark brown areas classified as VH for DPI and VL for uncertainty. Non-classified areas are identified in grey and were excluded based on a lack of available future resources or by constraints applied during the DPI analysis.

**Fig. 3:**
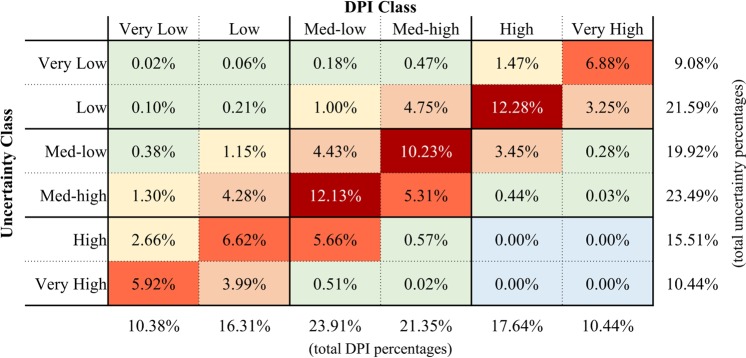
Cross tabular average percentages of development potential index (DPI) classes in each corresponding uncertainty class. Data for each DPI and uncertainty class were averaged across all 13 sectors and total percentages are summarized at the bottom (total percentage in DPI class) and right (total percentage in uncertainty class) of the table. Six colors classify percentages from lowest to highest (i.e., light-blue [0%], light-green [0–1%], yellow [1–3%], light-red [3–5%], red [5–7%], and dark-red [>10%]).

Similar to refs^39,120^, we classified all 13 CV datasets values into “Very Low” to “Very High” classes using the same DPI classification method based on z-score breakpoints^34^ (6 classes; Table 1). For each sector, we determined the DPI class(es) that exhibited the greatest uncertainty by calculating the percentages of DPI classes within each uncertainty class (for 36 different combinations; e.g., Fig. 2). We also averaged percentages of the 36 combinations across all 13 DPIs for a comprehensive uncertainty measure (Fig. 3). None of the DPI cells classified as “Very High” or “High” were found to fall within “Very High” or “High” uncertainty classes (Fig. 3). Rather, >99% “Very High” DPI cells and >75% of “High” DPI cells fell within the “Low” or “Very Low” uncertainty classes, respectively. These results indicate less uncertainty exists in high DPI areas. In contrast, the areas with highest uncertainty (i.e., “Very High” and “High” classes) fell within the “Low” and “Very Low” DPI classes for most sectors. These high uncertainty areas tended to occur in remote regions that lacked supporting infrastructure or market access but had higher than average resource potential (i.e., yield criteria in all DPIs), especially for the renewable and fossil fuel sectors. However, this same spatial pattern was not as prevalent for mining and agriculture sectors, where the highest uncertainty values largely occurred in regions that had low yield but were farther from markets or infrastructure. Readers can further explore the spatial distributions of uncertainty across each DPI by downloading data from figshare^34^.

#### Sensitivity analysis

For each sector DPI map, we evaluated the sensitivity of the multi-criteria weights using a one-at-a-time (OAT) method: where we incrementally modified weights within a range of values and then compared the modified and original (DPI_*Orig*_) outputs^9,41^. Following ref.^115^, we varied weights −20% to +20% of original weight value in increments of ±2% (*n* = 21 simulation runs), and determined the change in cell counts within five DPI value bins (i.e., >0.0–0.2, >0.2–0.4, >0.4–0.6, >0.6–0.8, >0.8–1.0). These five, equal interval bins were used to evaluate the sensitivity across the spectrum of continuous DPI values to distinguish changes in consistent value ranges across sensitivity runs while also reducing computing resources needed for a per pixel measurement. For each criterion and its bins, we calculated the average percent change in cell counts relative to the counts from DPI_*Orig*_ (Online-only Table 6). We additionally evaluated the spatial differences in outputs by calculating the cell-based correlations (Pearson’s *r*) between the modified DPI outputs and DPI_*Orig*_. Detailed sensitivity reports that included the percentage change per bin per simulation run for all sector criteria and the corresponding correlations are packaged with individual DPI data bundles^34^.

Unsurprisingly, the most sensitive criteria were the highest weighted ones and the least sensitive criteria were the lowest weighted ones across all DPIs. The most sensitive criterion was resource yield for all sectors, except for coal, for which mining density (equally weighted as coal resource yield) was the most sensitive. The least sensitive criteria were distance to railways or ports (CSP, PV, CG, UO, UG, NMM), distance to major roads (CO), distance to urban areas (Wind, Hydro), or distance to coal power plants (Coal). Overall, biofuels DPI exhibited the greatest sensitivity due to high shifts in the lowest bin (>0–0.2) (Online-only Table 6; see additional details below).

For most criteria and sectors, the lowest bin (>0.0–0.2) exhibited the greatest sensitivity, likely because it had the smallest overall frequency of cells within the DPI_*Orig*_, and thus the greatest percent changes. To focus on high development potential areas that are most influential to predicting land expansion areas, we examined the sensitivity exhibited within the top bins. For the second highest bins (>0.6–0.8), the most sensitive criterion within any sector-MCDA was coal mining density followed by hydropower resource yield. For the top bin (>0.8–1), the resource yield criterion for wind exhibited the greatest shifts. To further examine the sensitivity of these three criteria, we calculated the maximum and average absolute cell value change on a cell by cell basis associated with the sensitivity run having the greatest degree of change for all cell values and for cells with DPI_*Orig*_ values > 0.5. Even for the sensitivity runs with the greatest weight change (i.e., +20% or −20%), the maximum absolute cell change was less than 0.1 and the average absolute change was less than 0.05 across all cells and for DPI_*Orig*_ cells that were greater than 0.50 (Table 3, see Supplementary Fig. 1 for mapped example with Coal DPI).

**Table 3:** Sensitivity analysis results summarized as change in DPI values.

DPI Sector	Criteria with greatest variability in high DPI bins	Sensitivity run with greatest change	Maximum absolute change from DPI_Orig_ cell values	Average absolute change from DPI_Orig_ cell values	Maximum absolute change from DPI_Orig_ cell values > 0.5	Average absolute change from DPI_Orig_ cell values > 0.5
Coal	Active coal mining density	−20%	0.066	0.036	0.066	0.049
Hydro	Resource yield	−20%	0.091	0.039	0.091	0.023
Wind	Resource yield	−20%	0.087	0.026	0.087	0.023

Sector-specific maximum and average absolute change in cell values from DPI_*Orig*_ cells (all cells and cells >0.5) to DPI cells produced by the sensitivity run with the maximum weight change (i.e., +20% or −20%). Data are presented only for sectors that exhibited the greatest variability in the high DPI bins, i.e., 4 (>0.6–0.8) and 5 (>0.8–1.0). Abbreviations of sector are as follows: Coal – coal mining, Hydro – hydropower, and Wind – wind power.

Overall across all sectors, the binned value of most cells remained the same, and there were no cells that either increased or decreased more than one bin level from that of the original run. Furthermore, spatial correlations were *r* ≥ 0.971 for all sensitivity runs across all sectors, indicating low spatial variance in the DPI outputs due to modified weights. An overall low sensitivity was further reinforced by an only slight change (i.e., ~0.05) detected in cell values for the three most sensitive sector criteria when applying the maximum weight change.

## Data Records

For each development sector, three spatial datasets (i.e., the continuous DPI, the classified DPIs, and the classified uncertainty map) are accessible via figshare as GeoTIFF raster datasets at 1-km resolution using the Mollweide projection^34^. Due to large file sizes and for ease of access, all sector DPI data are bundled together within a correspondingly named zip file (e.g., Wind.zip). Each zip file contains: the continuous DPI raster dataset, the classified DPI raster dataset, the classified uncertainty raster dataset, all parameter descriptions and values (i.e., constraints, criteria correlations and weights, and AHP comparison values and matrix consistency ratio) used to produce the DPI, along with the full DPI sensitivity report. Additionally, four zip files (i.e., DPI_Inputs_and_Scripts_Part01-04.zip) are provided and contain all input data and Python scripts necessary to reproduce any DPI^34^. All three raster datasets per sector can also be viewed and examined interactively at http://s3.amazonaws.com/DevByDesign-Web/Maps/DPI_viewer/index.html.

## Technical Validation

To validate the DPIs, we used spatial point locations of planned or recently developed renewable energy power plants^123,124^, recent lease and claim boundaries identifying where fossil fuels and mining development is permitted^125,126^, and recent areas of crop expansion^127^. We compared mapped DPI classes to recent or planned development locations and determined the percentage of overlap and non-overlap (“none” class; Table 4).

**Table 4:** Spatial validation of DPI maps.

Recent and Potential Development Data	Data Type	Data Spatial Extent	Sample Size	DPI Overlap (% total)						
				Very high	High	Med- high	Med- low	Low	Very low	None
CSP Plants^123^	Points	North America	5	4 (80%)	0 (0%)	0 (0%)	0 (0%)	0 (0%)	0 (0%)	1 (20%)
PV Plants^123^	Points	North America	2,238	124 (6%)	488 (22%)	1,446 (64%)	115 (5%)	1 (0%)	0 (0%)	65 (3%)
Large PV Plants^123^ (i.e., capacity >=20 MW)	Points	North America	483	71 (15%)	173 (36%)	225 (47%)	13 (3%)	0 (0%)	0 (0%)	1 (0%)
Wind Farms^123^	Points	North America	411	175 (43%)	171 (42%)	37 (9%)	2 (0%)	1 (0%)	0 (0%)	25 (6%)
Hydropower^124^	Points	Global	2,231	457 (20%)	511 (23%)	637 (29%)	316 (14%)	169 (8%)	141 (6%)	NA
Large Hydropower^124^ (i.e., capacity >=30 MW)	Points	Global	946	284 (30%)	197 (21%)	233 (25%)	140 (15%)	51 (5%)	41 (4%)	NA
Coal Permits^126^	Polygons	US	10,848 km^2^	9,458 (87%)	805 (8%)	126 (1%)	0 (0%)	0 (0%)	0 (0%)	459 (4%)
Oil and Gas Leases^125^	Polygons	Western US	74,150 km^2^	32,304 (44%)	17,305 (23%)	18,404 (25%)	2,496 (3%)	120 (0%)	0 (0%)	3,521 (5%)
Mining Claims^125^	Polygons	Western US	42,392 km^2^	15,430 (36%)	14,601 (35%)	9,675 (23%)	2,209 (5%)	188 (0%)	51 (0%)	256 (1%)
Crop Expansion^127^	Pixels	Contiguous US	83,686 km^2^	23,819 (29%)	22,508 (27%)	15,399 (18%)	7,688 (9%)	5,941 (7%)	1,960 (2%)	6,371 (8%)

The percentage of overlap between mapped DPI classes and recent and potential development locations with information on data type, spatial extent, and sample sizes.

For solar power plants and wind farms, we used the most comprehensive database on recently developed or planned development locations^123^; data were only available for North America. We included records with available x/y coordinates (i.e., not city or county) for facilities constructed no earlier than 2016 or that were currently planned (see Table 4 for sample sizes). We assigned a DPI class value for each point based the closest DPI classified cell that fell within a maximum distance representing the square-root of the mean facility-area reported for the sector. For example, CSP plants average 6.64 km^2^ in size^128^, so we assigned the DPI class of the nearest cell within 2.58 km (i.e., square-root of 6.64), otherwise the location was classified as not having a class (i.e., None). We used a distance threshold of 1.77 km for PV based on ref.^128^ and 7.35 km for wind based on ref.^129^. We found that all but one of the CSP plants fell within the very high DPI class (Table 4). The vast majority of utility-scale PV power plants (92%) and all but 13 of the large PV plants (i.e., >=20 MW, cutoff identified by ref.^128^) fell within very high, high, or medium-high DPI classes (Table 4). Similarly, 85% of all wind farms fell within our mapped high and very high DPI areas and only twenty-five sites (6%) fell outside of any DPI class. For hydropower, we relied on the a dataset that identified proposed and currently constructed hydropower dams globally^124^. Because there were no feature-level location error assessments (i.e., how accurately each dam location was mapped), we only used future dam sites which were within 1 km of any DPI category cells. We found that 72% of all dam locations and 76% of large hydropower dams (i.e., >30 MW, cutoff identified by ref.^130^) fell within medium-high to very high DPI classes.

For coal, we used a U.S. coal mining permit database^126^ (n = 4,650 permits), that maps boundaries where companies have the right to disturb land for the mining and will be required by law to reclaim the site. Based on intersecting these lease boundaries with mapped DPI classes, we found that 87% of permit areas not mined fell within the highest DPI (only 4% were outside of any DPI cell). For oil and gas and mineral extraction sectors, we used a U.S. lease databases for oil and gas removal and mining claims^125^. Given the lack of publicly available data for these sectors and the high costs associated with more expansive proprietary data, we were limited to data within 10 western U.S. states: California, Oregon, Nevada, Idaho, Utah, New Mexico, Colorado, Wyoming, Montana, North Dakota, and South Dakota. Because oil and gas lease data did not distinguish the resource (i.e., oil or gas) or the method used (i.e., conventional or unconventional), we combined the DPI classes for CO, CG, UO, and UG and maintained the highest class per cell. We similarly combined metallic and non-metallic mining claims because these were not distinguished in the dataset. We overlapped oil and gas leases and mining claims with their associated combined DPI maps and found that >67% of oil and gas leases were located within very high or high DPI scores (only 5% were outside of any DPI cell), and 71% of mining claims not already mined overlapped with the two highest DPI classes (only 1% fell outside of DPI cells).

For cropland, we relied on spatial maps of annual cropland percentages per 1-km^2^ within the conterminous U.S. over the past 150 years^127^ and calculated the percentage of expansion for the most recent year of 2016 (i.e., we subtracted 2015 from 2016 percentages and selected only those cells with positive values). This identified over two million pixels (totaling 83,685.87 km^2^) with cropland expansion, which we overlapped with Crop DPI maps and calculated the total expansion per DPI class. We found that 73% of cropland expansion occurred in medium-high to very high DPI classes and over 46,000 km^2^ (56%) occurred in the top two classes. We were unable to find an analogous biofuels expansion dataset but assert that these results offer indirect support given that the (1) the cropland expansion dataset includes all biofuel crops, and (2) our biofuels DPI was created from the yield potential of a subset of crops and the same feasibility criteria (i.e., market accessibility and land supply elasticity) as the cropland DPI.

## Usage Notes

The DPI maps generated here provide some of the first globally consistent land suitability maps at a fine resolution (1-km) that depict the potential expansion for 13 major development sectors related to renewable energy, fossil fuels, mining, and agriculture. Our approach offers an advancement to other products by factoring in resource yield potential alongside multiple spatial factors that influence development siting using a spatial MCDA approach. It also advances the global mapping of multiple energy and extractive sectors that increasingly play a role in land use change^131^, but have been overlooked relative to agriculture or urban expansion^132,133^. Further, we examined the uncertainty and the sensitivity of each DPI and validated results with the best available known locations of recent or planned development: efforts rarely performed even for site-based or regional land suitability analyses^41^.

We acknowledge that our DPIs, like all global data, are inherently prone to inaccuracies, omissions, and inconsistencies in both their spatial features and attributes. While we used the best publicly available and current data for our analyses, input datasets were not always comprehensive in regional coverage (an issue that plagues all global analyses); however, with the provided Python code, each DPI can be easily updated as new data becomes accessible. For example, because only proprietary, global pipeline spatial data were available, our oil and gas DPIs (i.e., CO, CG, UO, UG) lacked this important criterion in the analysis and instead we relied on distance to existing oil and gas fields as a proxy that identifies where pipelines exist. Additionally, our DPIs do not consider governmental actions (e.g., environmental regulations, incentives, tax breaks) that often influence development siting, and may not capture land expansion under varying market changes and technological advancements. Given the frequency of policy and market changes, variations across administrative units, and the effort required to maintain such a database, incorporating the above was beyond the scope of this study, but future work, especially if focused on a smaller focal area, should seek to capture these criteria and/or conditions to update DPIs. We also do not account for climate change, which has been shown to redefine future crop yields^13^, and has the potential to inundate areas of current suitable land and/or supporting infrastructure, relocate population/demand centers of resources, modify precipitation or cloud cover patterns that can alter hydropower and solar resources^134,135^.

Finally, we recognize there are multiple uncertainties throughout any MCDA process (e.g., setting constraints, calculating spatial criteria values, and selecting criteria weights), and we only evaluated the uncertainty and sensitivity of one primary source (criteria weights). Nevertheless, our DPI maps offer more detailed and consistent global products on the relative (rather than the precise) suitability of lands for future development expansion. Although we produced DPI maps at a 1-km resolution, we do not recommend the use of these data at this resolution for local land-use planning or siting of development. We provide the DPI maps at this resolution (1) given its consistency with the input spatial feasibility metrics; (2) because it allows for the aggregation of data into comparable zones of analysis (e.g., countries, states/provinces, ecoregions, watersheds, etc.) that circumvent the modifiable areal unit problem often introduced with coarse resolutions^136^; and (3) because it maximizes the potential discernment of spatial heterogeneity in global development patterns^137^. While our validation results produced favorable support of our products, we emphasize that more localized or detailed spatial MCDA analysis should be performed using much finer resolution and higher accuracy data to fully resolve land suitability at 1-km pixel level. In addition, feedback should be solicited from local decision makers, industry representatives, and sector specialists to select regionally-tailored criteria factors and assigning their influence (weights) on development in the analysis.

**Fig. 4:**
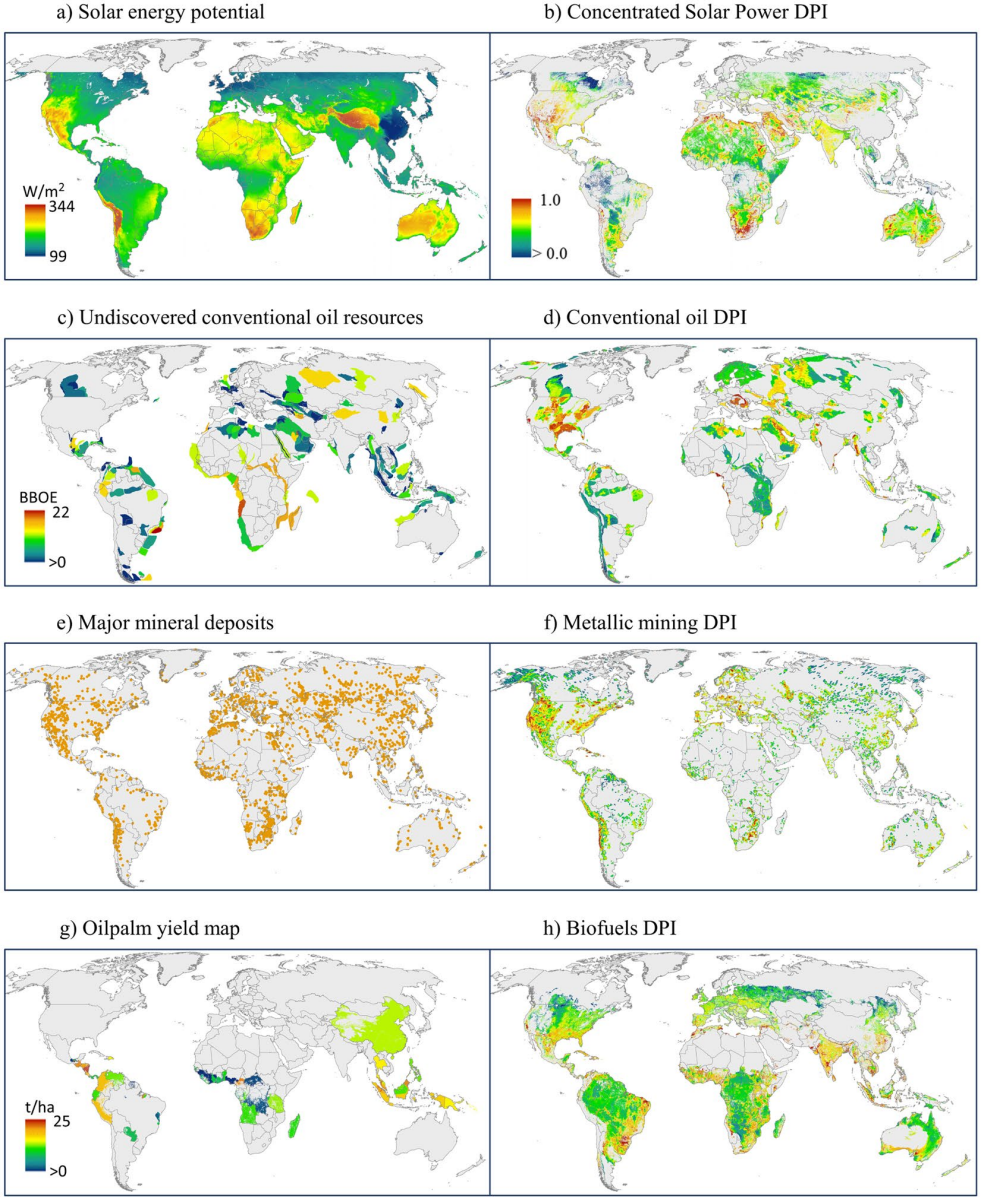
Comparison of DPIs with publicly available resource data. Data on resources were obtained from WRI Resource Watch and partners (left side panel) with the most analogous DPI maps produced by this study (right side panel). Color ramp for all maps are the same, with highest values in dark orange and lowest values in blue and null value in grey. Legend in first DPI map (**b**) can be applied to all other DPI maps (**d,f,h**). Map of only potential resource locations are displayed in a uniform orange color, i.e., large mineral deposit locations (**e**). Legend abbreviations for resource maps (**a,c,g**) are as follows: watts per square meter (W/m^2^), billion barrels of oil equivalent (BBOE), and tons per hectare (t/ha).

Despite these limitations, the timeliness and substantial need for these types of data are demonstrated by an increasing number of online portals hosting spatial data on human development pressure along with environmental features: e.g., the World Resource Institute’s (WRI) Resource Watch (http://resourcewatch.org), the World Wildlife Foundation’s (WWF) Sight (http://wwf-sight.org/explore), the European Commission Joint Research Center’s Digital Observatory for Protected Areas (DOPA) (http://dopa-explorer.jrc.ec.europa.eu/), the Global Forest Watch (http://www.globalforestwatch.org), the United Nation’s MapX (http://www.mapx.org/), the UN Biodiversity Lab (https://www.unbiodiversitylab.org/about.html), and the World Bank’s Spatial Agent (https://olc.worldbank.org/content/spatial-agent-tutorial), among others. We note that the development pressure datasets hosted on these online portals predominately focus on current development patterns, thus, they are limited to retrospective or current planning efforts. Of the select datasets that capture potential future expansion areas, they tend to map only areas of unexploited resources (e.g., resource yield proxies) without integrating spatial feasibility factors. Figure 4 displays some of the representative resource datasets from the above sources in comparison with the most analogous DPIs from this study. Previous existing maps identify locations of resources without resource value attribution (Fig. 4e) or captures resource yield potential but without spatial details on siting constraints (i.e., Fig. 4a vs Fig. 4b) and/or siting feasibility (i.e., Fig. 4c vs Fig. 4d). In addition, these maps often only capture one segment of a sector thereby neglecting the overall sector development pressures (i.e., Fig. 4g vs. Fig. 4h). While current development maps delineate regions susceptible to single-sector expansion over the long-term and can be used in basic binary overlay assessments^32^, they cannot be used in a gradient spatial analysis that differentiates among areas likely to undergo varying levels of development growth by multiple sectors in the near term.

In addition to elucidating potential individual sector expansion patterns, these DPIs can be combined to produce a cumulative development pressure metric at regional or global scales. While each DPI is sector specific, the relative index value is a measurement of development suitability based on multiple criteria on resource yield potential and development feasibility. Combing multiple DPIs provides a method for illuminating those lands that are suitable for multiple development sectors and thus have more pressures for being used. Although all DPIs have been scaled from 0–1, the distribution of these values may vary across sectors. Thus, to ensure equitability across all DPI values in a cumulative map, we recommend standardizing or classifying each DPI relative to an area of interest (e.g., global, regional, country) prior to this process.

**Fig. 5:**
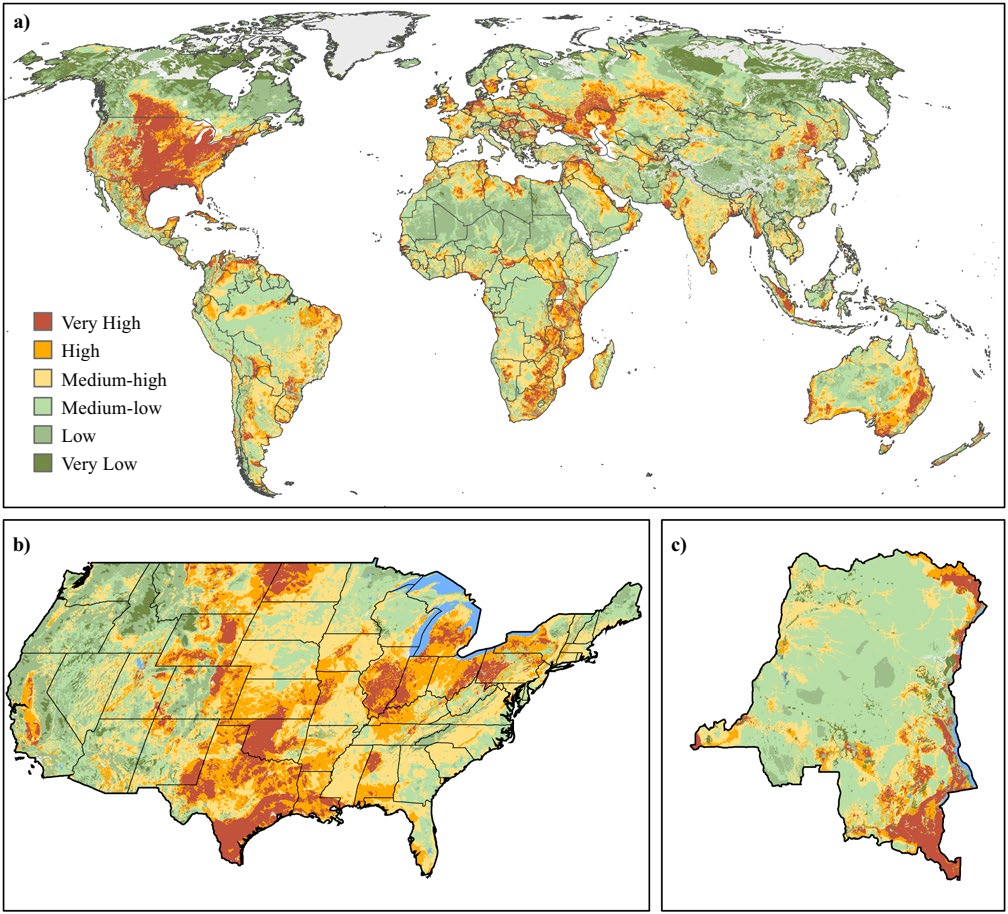
Global and regional-level cumulative development maps produced from standardized DPIs. Maps that display (**a**) global cumulative development potential map based on summing standardized global DPIs, and two regional-level cumulative development potential based on standardizing DPIs at the scale of the (**b**) United States (US) and (**c**) Democratic Republic of Congo (DRC). All maps use previously described z-score binning with legend in map (**a**) also applicable to maps (**b,c**).

Similar to a cumulative map of marine threats produced by ref.^138^, an additive approach is a simple and an effective way to create a cumulative DPI map. To provide an additive cumulative DPI map in line with the standardization guidelines we recommend above, one can apply globally, standardized z-score values for all continuous DPIs, which are equally-weighted and summed together (Fig. 5a). Such a map allows for countries, states, biomes, and/or ecoregions across the globe to be compared based on cumulative scores (i.e., prior to classification), thereby, identifying areas of varying levels of future development pressure. If the focus is at a more regional or country scale, each continuous DPI can be standardized to that spatial extent before summation. This approach helps to discern high development pressures that are less apparent when DPI values are globally standardized (Fig. 5b,c).

The DPI datasets provide relative measures of development suitability across the most comprehensive set of sectors, thus, serve as important tools that can help anticipate future development patterns at broad spatial scales from multiple sectors. The DPI maps can be combined with existing maps on current land use and land cover (e.g., global Human Modification map^139^, ESA CCI dataset^67^) to help assess opportunity costs and the potential for additional land conversion in a given region. In addition, estimates of production and consumption demand, which influence the likelihood of sector expansion, can also be considered for when quantifying how much potential land may be converted by multiple sectors within a given region (as done by ref.^140^). Together these data with the DPI maps can be used to proactively prioritize regions at a global scale to better plan for near-term tradeoffs among economic development, population growth, and the environment.

## Supplementary Information

### ISA-Tab metadata file

Download metadata file

### Supplementary information

Supplementary Information

## Data Availability

For DPI replication and integration of potential future data updates, we provide via figshare all Python scripts and spatial data necessary to replicate each DPI. Because these DPI scripts require input criteria data totaling 65 GBs in size, we bundled both scripts and data into four compressed files, DPI_InputsAndCode_Part01-04.zip^34^. These scripts use ArcPy, a Python module associated with ESRI’s ArcGIS Desktop software (www.esri.com) and require both the Advance license of this mapping software and an accompanying Spatial Analyst extension license. A *README.pdf* accompanies these zip files and provides all necessary instructions to setup the database and run any of the DPI scripts.

## Online-only Tables

**Online-only Table 1:** Literature review of sector development studies using spatial data and published within last ten years.

Source of Analysis	Year	Sector	Spatial Analysis			Spatial Constraints (exclusions)					Notes*
			*Type*	*Extent*	*Resolution*	*Biophysical**			*Land Use*	*Administrative*	(Other spatial factors used – or – spatial ranking descriptions)
						*Resource*	*Topography*	*Landcover*			
Bosch *et al*.^25^	2017	Wind	YP	Global	1-km	capacity factor (CF) < 15%	slope > ~11° (20%) elevation > 2000 m	irrigated croplands, wetlands, artificial surfaces, water, snow and ice		protected areas (PAs) all identified by World Database of Protected Areas (WDPA)	Land suitability refined by land cover types in Table 4.
Hoes *et al*.^18^	2017	Hydro	YP	Global	1-km	river discharge (Q) < 0.1 m^3^/s	<1-meter difference between two adjacent river cells (~255 meters at equator)				Gross theoretical potential based on global head and stream discharge calculations.
Dai *et al*.^22^	2016	Wind	YP $$	Global	1-km	NA (based on relative price of wind production in relation to other energy sources)	slope > ~31° (60%) elevation > 2000 m	water, wetlands, snow and ice	urban	PAs (no definition)	Land cover suitability scores listed in Table 2. Distance from urban areas used to measure energy loss and cost of transmission.
Eurek *et al*.^27^	2016	Wind	YP	Global	1-km	net CF < 26%	slope >20° (~36%) elevation > 2500 m	permafrost areas, snow and ice, water	urban	PAs (WDPA: IUCN Cats. I-III)	Landcover suitability scores listed Table 1. Distance from large power plants and large cities (proxy for transmission lines): 0-80 km – near, 80-161 – mid, >161 – far
Silva Herran *et al*.^23^	2016	Wind	YP $$	Global	10-km	NA	slope > 20° (~36%) elevation > 2000 m	water, wetlands, snow and ice	urban	PAs (no definition)	Identified wind potential within 3 ranges of urban areas 10 km, 20 km, 30 km.
Deng *et al*.^26^	2015	CSP	YP	Global	1-km	Direct normal irradiance (DNI) < 1900 kWh/m^2^/yr (~217 W/m^2^)	slope > 2° (~4%)	all forest and mix-forest, coast, cliffs, dunes, water, rock and ice	urban	Pas (Natura 2000 and WDPA: IUCN Cats. I–VI)	Land availability refined by land cover types identified in Table 2. Distance from infrastructure (defined in Fig. 1) used for three distance categories of very near, near, and far.
see above		PV	YP	Global	1-km	none	slope >15° (~27%)	see above	urban	see above	see above
see above		Wind	YP	Global	1-km	wind speed < 6 m/s	slope >15° (~27%) elevation > 2000 m	rain forest, tropical forest, coast, cliffs, dunes, water, rock and ice	urban	see above	see above
Eitelberg *et al*.^8^	2015	Crop	LS	Global	na	Literature review of constraints used in modeling potentially available croplands identified in Table 3					Only identifies suitable areas for agriculture without prioritization.
Köberle *et al*.^21^	2015	CSP	YP $$	Global	50-km	DNI < 1095 kWh/m^2^/yr (~125 W/m^2^)	none	forests, tundra, and wooded tundra	urban	bio-reserves (no definition)	Land availability refined by land cover types identified in Table 1. Applied cost based on distance from load centers (US$2,390,00/km).
see above		PV	YP $$	Global	50-km	none	none	see above	urban	see above	see above
Oakleaf *et al*.^20^	2015	Solar	LS	Global	50-km	Global horizontal irradiance (GHI)< ~ 1595 kWh/m^2^/yr (182 W/m^2^)	slope >3° (~5%)	water, wetlands, rock and ice, and artificial areas	urban and land > 80 km from existing roads	none	Solar and Wind LS produced by multiplying feasibility by suitability by resource raster datasets and summed multiplication within 50-km cell. Feasibility raster dataset produced by equal weighting distance to demand centers (1 closest -0.001 furthest) and distance to power plants (1 closest -0.001 furthest) all values within 5-km cells were averaged and then multiplied by 2 for countries with wind development. Suitability raster dataset produced from constraints placed in binary raster (1-suitable, 0 – excluded) summed per 5-km cell. Solar resource raster dataset produced from global horizontal irradiation values (1 highest – 0.001 lowest suitable i.e. 182 W/m^2^)
see above		Wind	LS	Global	50-km	wind speed < 6.4 m/s	slope > 20° (~36%)	water, wetlands, rock and ice, and artificial areas	urban and land > 80 km from existing roads	none	See notes above with wind resource raster dataset produced from wind speed map (1 highest - 0.001 lowest suitable i.e. 6.4 m/s)
see above		Coal	LS	Global	50-km	outside of coal-bearing areas	none	none	none	none	LS based on coal reserve estimates (i.e. million short tons) per 50-km.
see above		CO, CG	LS	Global	50-km	any geological province without either CO or CG estimated undiscovered resources	none	none	none	none	LS based on undiscovered COG reserve estimates of billion BOEs per geological province
see above		UO, UG	LS	Global	50-km	any shale/sediment formations without recoverable UO or UG	none	none	none	none	LS based on undiscovered UOG reserve estimates of billion BOEs per assessment area
see above		Mining	LS	Global	50-km	any 50 km^2^ area without an identified mineral deposit	none	none	none	none	LS based on mineral deposit counts per 50-km
see above		Ag	LS	Global	50-km	estimated agricultural expansion <= 0	none	none	urban 100% agriculture	none	LS based on mean agricultural expansion rate per 50-km
see above		Bio	LS	Global	50-km	estimated crop expansion = 0	none	none	urban 100% cropped	none	LS based on gallons of gasoline equivalent (GGE) per 50-km
see above		Urban	LS	Global	50-km	urban expansion probability <= 0	none	none	urban	none	LS based mean urban expansion probabilities per 50-km
Zhou *et al*.^19^	2015	Hydro	YP $$	Global	50-km	none	none	none	urban	PAs (WDPA identified)	Gross theoretical potential based on global head and stream discharge calculations.
Butt *et al*.^32^	2013	CO, CG	LS	Global	NA	any geological province without either CO or CG estimated undiscovered resources	none	none	none	none	CO and CG ranking based on total amount of future petroleum available per geological province. Used original geological province polygons. Identified coal basins for additional references of other fossil fuel development potential but didn't use in analysis.
Zhou *et al*.^24^	2012	Wind	YP $$	Global	1-km	none (due to goal of analysis)	elevation > 2000 m	wetland, water	urban	PAs (WDPA identified)	Three categories of land suitability refined by land cover types identified in SI Table 3. Calculated cost of building transmission based on Euclidian distance from transmission lines
Lu *et al*.^17^	2009	Wind	YP	Global	60 km × 50 km	CF < 20%	slope > ~11° (20%) elevation > 2000 m	forest, water, snow and ice	urban	none	Produced a global capacity factor map.
Hermann *et al*.^55^	2014	CSP	YP LS	Africa	28-km	DNI < 1800 kWh/m^2^/yr (~206 W/m^2^)	slope > 2° (~4%)	all forest and mix-forest, coast, cliffs, dunes, water, rock and ice	urban, cites, agricultural lands	PAs (WDPA: IUCN Cat. I–VI)	Suitability ranking based on DNI values (kWh/m^2^/yr): Suitable (1800 – 2000), Highly suitable (2000 – 2500), Excellent (2500 – 3000)
see above		PV	YP LS	Africa	28-km	GHI < 1000 kWh/m^2^/yr (~114 W/m^2^)	slope > 45° (~100%)	same as above	urban, cites	PAs (WDPA: IUCN Cat. I–VI)	Suitability ranking based on GHI values (kWh/m^2^/yr): Suitable (1000 – 1500), Highly suitable (1500 – 2500), Excellent (2500 – 3000)
see above		Wind	YP LS	Africa	9-km	Wind Speed < 4 m/s	slope > 45° (~100%)	rain forest, tropical forest, coast, cliffs, dunes, water, rock and ice	urban, cites	PAs (WDPA: IUCN Cat. I–VI)	Suitability ranking based on annual average wind speeds at 80 m (m/s): Limited (4-5), Suitable (5-7), Highly suitable (7-9), Excellent (>9)
Wu *et al*.^56^; Wu *et al*.^57^	2017, 2015	CSP	YP $$ LS	East Africa	5-km	DNI < ~ 2191 kWh/m^2^/yr (250 W/m^2^)	slope > ~3° (5%) elevation > 1500 m	forest, cropland, wetland, snow/ice, water (see Table 2 in Wu *et al*., 2015)	urban, population (pop.) density > 100/km^2^, railways and waterbodies and land up to 500 m from these features	PAs (WPDA identified) and lands within 500 m of PAs	2 km^2^ minimum developable area and 5 km^2^ project opportunity areas (POAs). For cost estimates see Table 7 in in Wu *et al*., 2015 Criteria maps at a resolution of 500 m and averaged criteria scores within POAs Criteria Values: See Table 8 in in Wu *et al*., 2015 Criteria Weights: Varies per country see online tables at http://mapre.lbl.gov/spatial-data/
see above		PV	YP $$ LS	East Africa	5-km	GHI < ~ 2453 kWh/m^2^/yr (280 W/m^2^)	see above	see above	see above	see above	see above
see above		Wind	YP $$ LS	East Africa	5-km	wind speed < ~ 6.2 m/s (300 W/m^2^)	slope > ~11° (20%) elevation > 1500 m	forest, wetland, snow/ice, water (see Table 2 in Wu *et al*., 2015)	see above	see above	see above
He & Kammen^58^	2016	CSP	YP	China	1-km	GHI < 1400 kWh/m^2^/yr (160 W/m^2^)	slope > ~2° (3%) elevation > 3000 m	forest, cropland, wetland, shurblands, savannas, grasslands, snow and ice	urban	PAs (WDPA identified)	Assessed YP based on two different grouping of constraints; upper (i.e. most available land for solar development or least restrictive constrains) and lower (i.e. least available land for solar development or most restrictive constrains), identified in Table 2. Capacity factors identified by province.
see above		PV	YP	China	1-km	see above	see above	see above	none	see above	
He & Kammen^60^	2014	Wind	YP	China	1-km	wind speed <= 6 m/s	slope > ~11° (20%) elevation > 3000 m	forest, cropland, wetland, water, snow and ice.	urban	PAs (WDPA identified)	Land availability refined by land cover types (Table 1 in ref) Slope % (0-2,2-3,3-4,4-20) varied power density (Table 1 in ref) Assessed YP based on two different grouping of constraints; upper (i.e. most available land for wind development or least restrictive constrains) and lower (i.e. least available land for solar development or most restrictive constrains), see Table 1 in ref
Lambin *et al*.^16^	2013	Crop	LS	Six regions /countries	varies	See Table 1 in ref. for listing of constraints. Constraint values dependent on regional location as described by 6 case studies.					
Lopez *et al*.^59^	2012	CSP	YP	United States	1-km	DNI < 1825 kWh/m^2^/yr (~208 W/m^2^)	slope > ~2° (3%)	water, wetlands	urban	PAs see Table A-4 in Ref. for PA list	Capacity factors for CSP based on DNI ranges (Table A-4)
see above		PV	YP	United States	1-km	none	see above	see above	urban	see above	State specific capacity factors for PV (Table A-2)
see above		Wind	YP	United States	1-km	wind speed < 6.4 m/s	slope > ~11° (20%)	water, wetlands plus land within 3km of wetlands	urban plus land within 3 km of urban	PAs plus land within 3 km of PAs see Table A-5 in Ref. for PA list.	
Mohammed & Alshayef^48^	2017	CO, CG	LS	Ayad, Yemen	NA	none applied					LS based on GIS, multi-criteria decision analysis (GIS-MCDA) using Analytical Hierarchy Process (AHP) for criteria weights and Weighted Linear Combination (WLC) to derive final LS used to prioritize COG development locations. Spatial criteria placed in three categories high, moderate, and low. Criteria and weights identified in Table VI. Validated spatially with existing oil and gas fields.
Jangid *et al*.^44^	2016	Wind	LS	Jodhpur District, India	not listed	average wind speed variation over months < 1.6 m/s at 20 m height	none	forested lands	including and within 500 m of residential land, > 1 km from a road	none	LS based on GIS-MCDA using AHP/WLC methodology to locate wind farms. Spatial criteria classified into low, medium, and high. Criteria (weight, highest category description): wind speed (0.4, highest), land use/cover (0.3, least and shortest vegetation), slope (0.15, flat), distance from roads (0.12, closest), distance from residential areas (0.03, furthest).
Baranzelli *et al*.^49^	2015	UO, UG	LS	Northern Poland	100-m	none (study area within shale gas basin)	none	caves and caverns, aquatic areas	urban and industrial areas, roads, railways, transmission lines, water wells, oil and gas wells	nature reserves, 100- year flood zones	LS based on GIS-MCDA using AHP/WLC methodology to site well pads. Spatial criteria continuous values identified in Table 4 and weights identified in Table 5. Analyzed two impact scenarios (high and low) for full development of resource. Used LS to place wells across landscape based on scenario definitions identified in Table 2.
Blachowski^47^	2015	Coal	LS	Southwest Poland	50-m	land outside coal deposits	none	none	none	none	LS based on GIS-MCDA using AHP/WLC methodology to rank highest conflict areas for coal mining. 15 spatial criteria and weighting identified in Table 4.
Brewer *et al*.^66^	2015	PV	LS	Southwest US	10-m	Global Tilted Irradiance (GTI) < 2373 kWh/m^2^/yr (~271 W/m^2^)	slope > 3.1° (~5%)	distances from rivers > 17.3 km	distances from roads > 0.56 km, distances from power lines > 32.7 km	none	Used constraints to restrict further analysis to two counties per state with highest area available for solar development. LS based on GIS-MCDA using WLC methodology to rank highest areas for utility PV development in selected Western US counties. Five spatial criteria withnine evenly distributed bins (i.e. 1–9); distance to roads (0–6 km), distance to rivers (0–45 km), distance to power lines (0–85 km), GTI (1095–2920 kWh/m^2^/yr), slope (0–90°) . Weights based on estimated cost differences identified Table 3. Created a public approval layer based on survey of acceptable distances (i.e. any, > 1 mile, > 6 miles, and >10 miles) from 5 features (i.e. residential areas, ag lands, cultural and historic areas, bird breeding and nesting sites, and recreation areas). Combined LS with public approval layer to identify suitable areas with the least public resistance.
Hernandez *et al*.^61^	2015	CSP	LS	California, US	30-m	DNI < 2190 kWh/m^2^/yr (~250 W/m^2^)	slope > ~2° (3%)	water, snow and ice	distances from roads >10 km, distances from transmission lines >20 km	areas where unlawful to build roads based on US and California statutes	LS based on compatibility index: used decision support tool, the Carnegie Energy and Environmental Compatibility (CEEC) model, to develop a three-tiered spatial environmental and technical compatibility index (i.e. Compatible, Potentially Compatible, and Incompatible). Land cover types impacted by PV and CSP solar identified in Table 1. Constraint listings for solar development base on older literature found in Table S4.
see above		PV	LS	California, US	30-m	DNI < 1460 kWh/m^2^/yr (~166 W/m^2^)	slope > ~3° (5%)	see above	see above	see above	see above
Zolekar & Bhagat^51^	2015	Ag	LS	Upper Pravara and Mula River Basin, India	5.8-m	NA	none – all slopes categorized in spatial criteria	water – all other land cover categorized in spatial criteria	none – land use categorized in spatial criteria	none	LS based on GIS-MCDA using AHP/WLC methodology to produce land suitability of agriculture in “hilly zones”. 12 spatial criteria categories and weights identified in Table 7. Criteria listings of various references for different types of land suitability identified Table 1.
Miller & Li^62^	2014	Wind	LS	Northeast Nebraska, US	200-m	wind speed < 5.6 m/s	slope > ~11° (20%)	wetlands, water	pop. density > ~58/km2 (150/mi2), >20 km from transmission line, >10 km from roads	none	LS based on GIS-MCDA using WLC with assigned criteria weights to produce land suitability for wind power development. Spatial criteria placed in 5 suitability categories (0/unsuitable – 5/high) Criteria and weights identified in Table 4: wind speed (0.25), distance to transmission lines (0.16), slope (0.16), land use (0.16), distance to roads (0.16), and pop. density (0.08).
Effat & Effat^46^		Solar	LS	Ismailia, Egypt	100-m	None	none	Water, wetlands, and sabkahs (i.e. salt flats)	urban areas and land within 2 km of urban areas, cultivated lands	none	LS based on GIS-MCDA using AHP/WLC methodology to produce a prioritization map for solar development. Spatial criteria placed into ten categories identified in Tables 6-7. Criteria (weights, highest category description): solar radiation (0.47, highest), aspect (0.24, southern) distance to powerlines (0.12, closest), distance to roads (0.09, closest), and distance to cities (0.08, closest)
Elsheikh *et al*.^63^	2013	Ag	LS	Terengganu, West Malaysia		based on crop type selected by user within tool					LS based on GIS-MCDA using the Agriculture Land Suitability Evaluator (ALSE) specific for tropical and subtropical crops. Spatial criteria created for each crop in tool and placed into five suitability levels typical for ag suitability (i.e. S1, S2, S3, N1. and N2)
Gorsevski *et al*.^64^	2013	Wind	LS	Northwest Ohio, US	30-m	none	none	wetlands, water	developed areas, airports	none	LS based on GIS-MCDA using WLC for combining spatial criteria and weights Borda ranking method for deriving weights Spatial criteria continuous from 0-1 identified Table 1. Weights identified in Table 2. Performed spatial sensitivity on weights.
Pazand *et al*.^50^	2011	Mining	LS	Northwest Iran	100-m	none applied					LS based on GIS-MCDA using AHP/WLC methodology to produce a prioritization map for copper porphyry exploration. Used five main spatial criteria; airborne magnetic, stream sediment geochemical data, geology, structural data and alteration zones. Criteria weights identified in Table 6.
Clifton & Boruff^65^	2010	CSP	LS	Western Australia	90-m	DNI < 2000 kWh/m^2^/yr (~228 W/m^2^)	slope > ~2° (4%)	forest, wetland, snow/ice, water (specifics identified in Table S2)	none	PAs (no definition), cultural sites	Development potential classes based on CSP index standard deviations from the mean: high (>2), medium (1–2), low (0–1). Criteria Values to produce CSP index: Ag productivity (0 – highest yield to 1 lowest yield): 0.16 Distance to roads (1 – closest to 0 furthest, no threshold distance): 0.16 Distance to transmission lines and substations (same as roads): 0.16 DNI values (1 max to 0 lowest): 0.5
Janke^45^	2010	CSP	LS	Colorado, US	1500-m	none	none	none	none	all US federally managed lands (due to goal of study)	LS based on GIS-MCDA using WLC with assigned weights. Spatial criteria and weights identified in Table 1.
see above		Wind	LS	Colorado, US	1500-m	none	none	none	none	see above	see above
Khoi & Murayama^53^	2010	Crop	LS	Tam Dao National Park Region, Vietnam	30-m	none (used fuzzy spatial criteria with 0 values but no exclusions related to overall suitability scoring)					LS based on GIS-MCDA using AHP/WLC methodology to produce a crop farming suitability map. Used method to derive 3 suitability maps relating to terrain and water, soil quality, and access to roads and park. These three suitability maps were then applied weights using AHP and combined using WLC to produce final suitability. Spatial criteria had continuous value ranging from 0-1 identified in Table 2. Weights produced from AHP identified in Table 3.

Three types of analysis were reviewed and can be classified as land suitability (LS), yield potential (YP) of a resource, or economic feasibility ($$) of siting. Studies ordered by spatial extent analyzed from global to local and sub-ordered by date of reference. Abbreviations of development sector are as follows: Ag – agricultural expansion (undefined definition or combination of crop and pasture expansion), Bio – crop expansion specific to biofuel crops, Coal – coal mining, CO – conventional oil, CG – conventional gas, Crop – crop expansion, CSP – concentrated solar power, Hydro – hydropower, Mining – mineral extraction, PV – photovoltaic solar power, Solar – solar power without specification of technology, UO – unconventional oil, UG – unconventional gas, and Wind – wind power. All values denoted with tilde symbol (~) indicate values were converted from the referenced value within the cited literature.

*All table and figure numbers identified in the Notes and Biophysical columns are found within the corresponding source document.

**Online-only Table 2:** Data sources, constraint thresholds, and processing steps to produce constraints and resource yield criteria maps.

Sector	Data sources (*n*; units; resolution)	Constraints (exclusions)^*^	Data layer(s) processing steps^†^
CSP	**Average annual direct normal irradiance (DNI)**^141^ (W/m^2^∙y; 3.7 km)	<125 W/m^2^∙y based on ref.^26^	i.→ For every *i*th cell calculated resource potential in MWh/km^2^ as *PD* ∙ *CF*_*i*_ ∙ 8760, where *PD* = power density of 17 MW/km^2^ ^56^, *CF*_*i*_ = spatially explicit capacity factor using DNI values and assuming 6-hr storage based on ref.^56^, and 8760 = number of hours in a year. ii.→ Limited analysis cells by constraints including removal of any operating CSP plants.
	Slope^142^ (%; 90 m)	>5% based on ref.^57^	
	Landcover^143^ (1 km)	wetlands, rock/ice, artificial areas, forested areas based on ref.^26^	
	CSP plant locations from SolarPACES^144^ (*n* = 96)^**§**^	any operating plants	
PV	**Average annual global horizontal irradiation (GHI)**^141^ (W/m^2^∙y; 3.7 km)	NA	i.→ For every *i*th cell calculated resource potential in MWh/km^2^ as *PD* ∙ *CF*_*i*_ ∙ 8760, where *PD* = power density of 30 MW/km^2^ ^56^, *CF*_*i*_ = spatially explicit capacity factor using GHI values and based on eq. 1 in. in ref.^56^ (while similarly assuming 4% outage rate and 4% inefficiency rate), and 8760 = number of hours in a year. ii.→ Limited analysis cells by constraints.
	Slope^142^ (%; 90 m)	>30% based on ref.^26^ ^‡^	
	Landcover^143^ (1 km)	wetlands, rock/ice, artificial areas, forested areas based on ref.^26^	
Wind	**Average annual wind speed** at 80 meters^145^ (m/s; 5 km)	<6 m/s based on refs^26,56,60^	i.→ For every *i*th cell calculated air density factor (AD_*i*_) by dividing air density (ρ_*i*_) by sea-level density of 1.225 kg/m^3^, where for each elevation (Z_*i*_): ρ_*i*_ = 1.225–1.94 * 10^−4^ * Z_*i*_. Values of *AD*_*i*_ ranged from 1.00 at sea level to 0.475 at 3,000 m elevation. ii.→ Calculated resource potential in MWh/km^2^ as *PD* ∙ *CF*_*i*_ ∙ *AD*_*i*_ ∙ 8760, where *PD* = power density of 5 MW/km^2^, *CF*_*i*_ = spatially explicit capacity factor based fitting a local polynomial regression (loess) to average annual wind speed data reported by ref.^56^, *AD*_*i*_ = air density factor calculated as described above, and 8760 = number of hours in a year. iii.→ Limited analysis cells by constraints including removing cells with ≥3 existing turbines.
	Slope^142^ (%; 90 m)	>30% based on ref.^26^^‡^	
	Elevation^146^ (m; 1 km)	>3,000 m based on ref.^60^	
	Landcover^143^ (1 km)	wetlands, rock/ice, artificial areas based on refs^23,26^	
	Wind turbine locations^**^ (*n* = 90,106)^**§**^	any cell with ≥3 turbines based on refs. ^59,147,148^ indications of wind development	
Hydro	**Hydropower potential locations**^18^ (*n* = 11,839,398; kWh/y)	cells with <1 MW potential to accommodate utility-scale hydropower development^130^	i.→ Limited hydropower potential locations to those generating ≥8,760,000 kWh (i.e., 1 MW) and removed any locations ≤1 km of existing hydroelectric dams, consistent with average distance of existing dams (1.14 km; this study based on GRanD data). ii.→ Hydropower potential locations spanned fully inundated, or partially inundated cells; attribute values of fully inundated cells to the closest terrestrial cell (*n* = 935). iii.→ Divided kWh by 1000 to calculate MWh and rasterize locations.
	Existing hydropower dams^149^ (*n* = 2,134)^**§**^	any cell within 1km of a dam identified as hydroelectric for main, major, or secondary use	
Coal	Global **coal basins** maps^20^ (*n* = 2,053)	NA	i.→ For each jurisdiction (country or state): a.→ Clipped basins by jurisdiction and calculate basin area within b.→ Divided estimates of technically recoverable coal by the area of the coal basins within the jurisdiction to obtain million short ton/km^2^ c.→ Rasterized basins and attribute million short ton/km^2^ to every coal basin cell within the jurisdiction. ii.→ Merged jurisdictional results into a single raster of coal yield. iii.→ Limited analysis cells by constraints including removal of any cells with existing active coal mine.
	Country or state-level estimates of **technically recoverable coal**^20^ (*n* = 305; million short tons)	NA	
	Existing coal mines from 8 datasets^150–157^ (*n* = 2,301)^**§**^	any cell with active coal mine	
CO	Global^158,159^, U.S.^160^ and Australia^161^ **assessment units (AUs)** of median recoverable oil reserve (*n* = 778; barrels of oil or bbl), and dry gas (n = 705; ft^3^), and liquid natural gas (NGL) (*n* = 823; bbl)	NA	i.→ Geo-referenced and digitized all world shale prospective area (PA) maps and assigned technically recoverable values to each PA. ii.→ For dry gas, converted reserve estimates from ft^3^ of dry gas to BOE using a conversion factor of 6000 ft^3^ to 1 BOE, then summed for each PA or AU the total gas estimates in BOE from the converted dry gas and NGL. iii.→ For each sector AU or PA: a.→ Divided the technically recoverable oil or gas reserve estimates by the area of the AU/PA to obtain BOE/km^2^. b.→ Rasterized AU/PA and attribute BOE/km^2^ to every cell. iv.→ Combined all AUs/PAs for each sector, summarizing overlapping BOE/km^2^ cell values.
CG			
UO	World shale **prospective areas (PAs)**^162^ risked recoverable oil (*n* = 109; bbl) and gas (*n* = 192; ft^3^), and U.S. unconventional **AUs** of median recoverable oil reserves^163,164^ (*n* = 22; bbl), dry gas (n = 130; ft^3^), and liquid natural gas (NGL) (*n* = 113; bbl)	NA	
UG			
MM	**Mineral deposit point locations** and categorical deposit sizes (e.g., very larger, large, medium) from refs^165–172^ combined into a global dataset consisting of 167 minerals (*n* = 320,843)^††^	84 metallic minerals (e.g, gold, silver, iron), plus gemstone and uranium (*n* = 207,258)^††^	i.→ For any given mineral, removed spatial duplicates and assign highest deposit value to deposit location^§§^. ii.→ Split data to U.S. and non-U.S. regions^‡‡^. iii.→ Ran Kernel Density (KD) using: a.→ Radii of 60 km for metallic, as used for gold deposits in Australia^75,76^, and 20 km for non-metallic, as used for aggregate minerals in Poland^173^. b.→ Weights based on classifications and criteria in ref.^74^ as follows: 1 – occurrences, 4 – small deposits, 9 – medium deposits, 16 – large deposits, and 25 – very large deposits (volume classifications in Appendix 1 of ref.^74^). iv.→ Selected only cells with KD > 0.001 deposits per km^2^ based on ref.^76^. v.→ Limited to analysis constraints where we defined cells with existing mines as any cell containing past and current mines in the collated database, or cells with ≥ 50% overlap with mapped mineral or industrial areas^**^. vi.→ Standardized and merged U.S. and non-U.S. regions into one global map.
NMM		83 non-metallic minerals (e.g., aggregates, gravel, and sand; *n* = 113,498)^††^	
Crop	**2012 yield and area data by political unit** (national or sub-national)^174^	Included barley (*n* = 5,389), cassava (*n* = 6,053), maize (*n* = 13,681), oil palm (*n* = 2,007), rapeseed (*n* = 3,496), rice (*n* = 3,984), sugarcane (*n* = 2,912), sorghum (*n* = 5,421 soybean (*n* = 5,707), and wheat (*n* = 7,377)	i.→ For each political unit, obtained the area-weighted average yield in ton/km^2^ (response variable). ii.→ Processed explanatory variables (*abbreviations*): a.→ Used WORLDCLIM to calculate crop-specific annual growing degree days (*GDD*) and mean annual precipitation (*Prec*) following ref.^79^ and converted temp of coldest month into a binary variable of 1 if coldest month of the year is between −8 °C and +5 °C, else 0 (*VF)*. b.→ Used WATCH input data to calculate number of days with temp >34 °C (*KDD34*). c.→ Used Harmonized World Soil Database V1.2 slope datasets to identify percent of 10 km cell greater than 10% and 30% (*slgt10* and *slgt30*). d.→ Used ISRIC world soil information database to extract the soil water holding capacity for top 20 cm (*awc*). e.→ Used irrigation fraction data to linearly extrapolate data for 2000 and 2005 while constraining fraction 0–1 (*Irr*). iii.→ For each crop, modeled ton/km^2^ as a function of climate, slope, soil, and irrigation data using a 95^th^ percentile quantile regression on standardized variables. Started with general model: *GDD+Prec+ Irr+VF + KDD34+slgt10+slgt30 +awc +GDD*^*2*^*+Prec*^*2*^*+GDD*Prec+ Prec*Irr* and conducted backward selection with bootstrapping methods^†††^ to select crop-specific models and coefficients (Table 3). iv.→ For every crop, created global predictive maps using model results: a.→ Used input explanatory variables data and remove cells with values <2.5^th^ and >97.5^th^ percentile CIs based on model coefficient results. b.→ Predicted global yield map based on model coefficients. c.→ Removed any cells with predicted values < the 10^th^ percentile observed area-averaged yield (ton/km^2^). v.→ Limited analysis cells by constraints.
	**WORLDCLIM**^175^ (1-km)	Thornethwaite Moisture Index <0.25 without irrigation fraction >0.05	
	**Water and Global Change (WATCH) Forcing Data**^176^ (50-km)	NA	
	**Slope**^142^ (%; 90 m)	>30% based on ref.^8^	
	**Average water holding capacity**^177^ (10 km)	NA	
	**Irrigation fraction**^178^ (10 km)		
	Percent cropland^179^ (250 m)	Cropland ≥ 95%^180^	
Bio	see above	see above	i.→ Used cropland yield values (tons/km^2^) derived above for the five first generation biofuel crops (listed below) and multiplied yield by gallon of gasoline equivalent (GGE) conversion rates^20^ (maize-162.47, oil palm-56.55, rapeseed-99.41, sugarcane-32.1, soybean-46.41). ii.→ Limited analysis cells by constraints identified in crop expansion above.

Table includes references, justifications, and rational for producing resource yield layers and constraints for the 13 development sectors. Data sources column identifies data used in yield estimates in **bold** (e.g., **Average annual direct normal irradiance**) and includes data on the number of points or polygons used for yield estimates and/or constraint mapping (*n*), original input units provided by data (units), and cell size of raster data (resolution). Constraints column identifies the threshold value of the data source used to map non-suitable lands that were excluded from each development potential index (DPI). Abbreviations of sector are as follows: CSP – concentrated solar power, PV – photovoltaic solar power, Wind – wind power, Hydro – hydropower, CO – conventional oil, CG – conventional gas, UO – unconventional oil, UG - unconventional gas, MM – metallic minerals, NMM – nonmetallic minerals, Crop – cropland expansion, Bio – biofuels expansion.

^*^Urban areas defined as human-built environments created by ref.^181^ were excluded from resource yield maps for all sectors.

^†^Online-only Table 1 provides a detailed literature review that informed parameter selection and/or threshold values.

^§^Point locations of existing development used for excluding cells from resource yield maps.

^‡^Reference^26^ used a slope of 27%, however because the Harmonized World Soil slope data used here, which were derived from 90-m resolution digital elevation data, limit users to binned slope breaks (e.g, 2, 5, 10, 15, 30), we used the next closest bin break of 30%.

^**^Data from OpenStreetMap.org (©OpenStreetMap contributors, CC-BY-SA https://www.openstreetmap.org/copyright).

^††^87 locations had undefined mineral type and were therefore removed from the analysis; these data were used to derive resource yield and final mining DPIs based on kernel density analyses specified in processing steps.

^§§^To avoid inflating kernel density values, we removed spatial duplicates for each mineral (e.g., gold, sand, etc.) and assigned the highest deposit value to that location. We acknowledge that density values will still be higher if the same location was sampled for different minerals, or if multiple locations in close proximity were sampled for a given mineral. However, higher density values in these cases are justified as it is more likely that these areas will be developed given sampling intensity of the deposits.

^‡‡^82% of deposit locations was located within the U.S., hence we created mining density maps separately for the U.S. and non-U.S. regions, which we later normalized separately and then merged for a final global map of resource yield potential.

^†††^We used the built-in confidence intervals (CIs) in R (using rq() with the ci = TRUE option) unless either the CI upper and or lower bounds were numerically infinite. In this case, we determined CIs with a bootstrap method by constructing an NxM array of parameters where M is the number of parameters and N is the number of bootstrap samples (using sampling with replacement). The 2.5% and 97.5% quantiles of the M values in the distribution of each parameter represent the lower and upper bounds. We used N = 200 when determining the least significant parameter in the model simplification step discussed above, and we used N = 1000 when determining confidence intervals.

**Online-only Table 3:** Selected crop-specific yield models and coefficients.

Crop	Variable Coefficients												
	Constant Term	GDD	Prec	Irr	VF	KDD34	slgt10	slgt30	awc	GDD^2^	Prec^2^	GDD*Prec	Prec*Irr
Barley	6.649962	−2.86163	1.956107	0.211724	ns	ns	0.367577	ns	ns	xxx	−1.207921	−1.962384	−1.283324
Cassava	19.16283	0.666753	−7.958917	8.454535	ns	−5.187399	ns	ns	ns	4.660226	ns	−7.958917	ns
Maize	8.248736	−2.447709	0.365055	0.680522	ns	ns	ns	−1.132499	ns	ns	ns	−0.890061	ns
Oil palm	17.124405	ns	ns	ns	ns	ns	ns	5.247929	ns	ns	ns	0.070376	ns
Rapeseed	2.812388	ns	−0.837681	xxx	0.509829	xxx	−0.852301	−0.900022	0.0194	−0.456553	0.07705	ns	0.043222
Rice	7.265925	−0.767782	−1.664991	0.259963	0.228184	−1.067667	ns	ns	ns	ns	0.192307	ns	0.06965
Sorghum	6.3906	−3.320566	0.343852	1.197672	0.223015	ns	ns	ns	0.119473	ns	ns	−1.490304	ns
Soybean	3.538115	−0.480592	ns	0.180917	ns	ns	ns	ns	ns	−0.249019	0.011759	−0.17576	−0.494005
Sugarcane	81.3797	−28.75697	ns	ns	−12.95342	−21.70054	ns	ns	0.306826	8.925447	5.231338	−23.47809	8.766958
Wheat	6.432947	−1.893532	1.601218	0.186331	0.556901	ns	ns	−1.23728	ns	ns	0.149179	−1.310299	−1.15477

Cells with value of “ns” indicated variable was not selected for corresponding crop-specific yield model. Abbreviations are as follows: GDD - annual growing degree days, Prec - mean annual precipitation, Irr - fraction irrigated, VF - temp of coldest month in a binary variable of 1 if coldest month of the year is between −8 °C and +5 °C or 0 if not, KDD34 - number of days with temp >34 °C, slgt10 - slope datasets to identify percent of 10 km cell greater than 10%, slgt30 - slope datasets to identify percent of 10 km cell greater than 30%, awc - soil water holding capacity for top 20 cm.

**Online-only Table 4:** Feasibility criteria used to map development potential. Criteria are listed with their relevant sectors, data sources, and spatial processing steps. Input data sample sizes (*n*), units, and spatial layer resolution, are specified as applicable. Abbreviations of sector are as follows: CSP – concentrated solar power, PV – photovoltaic solar power, Wind – wind power, Hydro – hydropower, Coal – coal mining, CO – conventional oil, CG – conventional gas, UO – unconventional oil, UG - unconventional gas, MM – metallic minerals, NMM – nonmetallic minerals, Crop – cropland expansion, Bio – biofuels expansion. All distant-based feasibility criteria calculated Euclidian distances from identified features using the World Two Point Equidistant project coordinate system and applied a Gaussian distance decay function to these distance values with a threshold distance (*h*) of either 100 km for renewable sectors (i.e., CSP, PV, Hydro, Wind) or 50 km for fossil fuel and mining sectors (i.e., Coal, CO, CG, UO, UG, MM, NMM) to derive final standardize criteria values from 0–1.

Feasibility criteria	Sectors	Data and sources (units; resolution as applicable)	Data layer(s) processing steps
Distance to major roads	CSP, PV, Wind, Hydro, Coal, CO, CG, UO, UG, MM, NMM	OpenStreetMap (OSM)^*^ highway:motorway, highway:trunk, highway:primary, highway:secondary (*n* = 6,881,049)	i.→ Selected major road categories of motorway, trunk, primary, or secondary. ii.→ Calculated distance from major roads and standardized distance values with sector-specific Gaussian function.
Distance to railways or ports	CSP, PV, Wind, Hydro, Coal, CO, CG, UO, UG, MM, NMM	OSM^*^ railway:rail (*n* = 1,537,71)	i.→ Combined railway datasets into a single linear feature dataset using only DCW data not within 1 km of OSM railway features. ii.→ Selected seaports supporting cargo vessels >500 feet. iii.→ Calculated separate distance surfaces for railways and ports. iv.→ Selected per cell the minimum distance of the two distance surfaces and standardized distance values with sector-specific Gaussian function.
		Digital Chart of the World (DCW)^182^ railroads (*n* = 194,261)	
		World Port Index^183^ (*n* = 1,135)	
Electricity accessibility	Coal, CO, CG, UO, UG, MM	OSM^*^ power:lines (*n* = 514,785)	i.→ Combined transmission line datasets into a single linear feature dataset using only DCW data not within 1km of OSM powerline features. ii.→ Selected hydroelectric dams and power plants generating ≥ 1 MW of power. iii.→ Selected only lighted cells with DN values ≥ 12 similar to ref.^184^. iv.→ Calculated distance to the 3 input features (power lines, power plants and nighttime lights). v.→ Selected per cell the minimum distance across all 3 distance surfaces and standardized minimum distance values with 50-km Gaussian function.
		DCW^182^ power transmission lines (*n* = 103,419)	
		Hydropower dams^149^ (*n* = 2,134)	
		Power Plants^185^ (*n* = 50,968)	
		2013 stable nighttime lights^186^ (1 km)	
Distance to electrical grid^†^	CSP, PV, Wind, Hydro	OSM^*^ powerlines (*n* = 514,78)	i.→ Combined transmission line datasets into a single linear feature dataset using only DCW data not within 1km of OSM powerline features. ii.→ Selected only primary hydroelectric dams and power plants with ≥200,000 MWh/y generation, a power output that is commonly connected to national electrical grids^27^. iii.→ Calculated distance to the 2 input features (power lines and utility-scaled power plants). iv.→ Selected per cell the minimum distance of the two distance surfaces and standardized minimum distance values with 100-km Gaussian function.
		DCW^182^ power transmission lines (*n* = 103,419)	
		Hydropower dams^149^ (*n* = 1,540)	
		Power Plants^185^ (*n* = 9,044)	
Distance to urban areas	CSP, PV, Wind, Hydro	Urban areas^181^ (500 m)	i.→ Summed estimated 2015 population counts^187^ per km^2^ that fell within connected cells of urban areas (i.e., urban regions) identified as any human-built environments by ref.^181^. ii.→ Selected only urban regions with ≥ 50,000 people, a definition of urban by ref.^188^. iii.→ Augmented selected urban regions by adding any excluded urban region which contained a population place location with ≥ 50,000 people (*n* = 2,411 urban regions added). iv.→ Calculated distance from urban regions and standardized minimum distance values with 100-km Gaussian function.
		Global population places^189^	
Landcover	CSP, PV, Wind	Landcover^190^ (300 m)	i.→ For solar: a.→ Assigned scores to landcover codes from 1 (low development cost) to 3 (high development cost) based on ref.^56^, and a score of 4 for land cover classes excluded from ref.^56^ indicating the highest cost for development. b.→ Inverse scaled scores from 0‒1; i.e., criteria value (landcover codes): 0.00 (10‒90, 160‒190, 210‒220); 0.34 (100, 120‒122); 0.67 (110, 130, 140); 1.00 (150‒153, 200‒202). ii.→ For wind, averaged landcover scores from refs^25,27^ and assigned criteria values to associated landcover codes, i.e., criteria value (landcover codes): 0.00 (20, 160‒190, 210‒220); 0.10 (50‒61, 71‒90); 0.30 (62); 0.45 (30); 0.50 (120‒122); 0.505 (100); 0.65 (110,130,140); 0.7 (10–12); 0.75 (40); 0.85 (150–153); 0.9 (200–202). ii.→ For solar and wind: a.→ Resampled assigned landcover values to 1-km using average function b.→ Max-normalize averaged values.
Inverse population density	Wind, Hydro	United Nations-Adjusted Population Density for 2015^187^ (1 km)	i.→ For hydropower, calculated the inverse of the mean population density in a 3 × 3 km moving window. (based on median hydropower reservoir area of 10 km^2^ form data in ref.^149^). ii.→ For wind, calculated the inverse of the mean population density in a 6 × 6 km moving window (based on median wind farm size in US of 38 km^2^ ^129^). iii.→ For hydropower and wind and following ref.^56^, calculated the inverse density for values ranging 0–100 people/km^2^ and max normalized values to 1–0; all other density values (i.e., >100 people/km^2^) were assigned a value of 0.
Distance to producing oil and gas fields	CO, CG, UO, UG	Current and future oil and gas fields as of 2003^191^ (*n* = 1,273)	i.→ Selected field polygons with known development of either oil or gas (*n* = 1,005). ii.→ Supplemented selected field polygons with any fields overlapping with identified gas flaring (n = 25). iii.→ Created binary raster identifying selected fields. iv.→ Augmented raster with any 1-km cell with gas flaring occurrences. v.→ Calculated distance from fields and standardized distance values with 50-km Gaussian function.
		Gas flaring captured from night imagery 2006^192^ (1 km), 2012^193^ (1 km), 2016^194^ (500 m)	
Market accessibility	Crop, Bio	Accessibility to Cities^195^ (1 km; travel time in minutes)	i.→ Used market accessibility index formula described by ref.^196^ for large markets (i.e., 2 hour inflection point) and applied to accessibility to cities dataset.
Land supply elasticity	Crop, Bio	2013 stable nighttime lights^186^ (1 km)	i.→ Calculated percent of cropland expansion in US from 2010 to 2016^127^ per light intensity (i.e., DN values) categories of 0, >0–12, >12 as defined by^184^ as the DN value identifying the agricultural land/urban transition value. ii.→ Results identified 67% cropland expansion without lights (i.e., 0 DN) and 10% found in DN values >12. iii.→ Assigned unlit areas (DN 0) as 1, cells with DN values >0–12 as 0.5, and 0.0 for cells with values >12
Distance to aggregate demand centers	NMM	United Nations-Adjusted Population Density for 2015^187^ (1 km)	i.→ Selected only cells with ≥ 77 people/km^2^, or density identified by ref.^197^ which majority of aggregate development occurs. ii.→ Calculated distance from selected cells and standardized distance values with 50-km Gaussian function.
Distance to coal power plants	Coal	Power Plants^185^ (*n* = 6,684)	i.→ Selected power plants using coal combustion as defined by ref.^185^. ii.→ Calculated distance from power plants and standardized distance values with 1,300-km Gaussian function, an average haul distance for coal in the U.S.^198^.
Active coal mine density	Coal	Existing coal mines from 8 datasets^150–157^ (*n* = 2,301)	i.→ Merged 8 coal mine databases and select only current mines. ii.→ Ran kernel density using a 40-km radius, or half the threshold distance of coal mine concentration in Germany^199^. iii.→ Removed KD values ≤1 mine/km^2^. iv.→ Standardized remaining values (ranging 1–159) by: reassigning top 1% outliers the value of the 99^th^ percentile, log-transforming, and max-normalizing the data such that resulting values ranged 0–1.

^*^Data from OpenStreetMap.org (©OpenStreetMap contributors, CC-BY-SA https://www.openstreetmap.org/copyright).

^†^Distance to electrical grid is different from electricity accessibility in that distance to the electrical grid focuses on distributing produced energy whereas distance to electricity accessibility focuses on proximity to any electricity source.

**Online-only Table 5:** Criteria weight ranges applied within the uncertainty analysis for all 13 development potential indices (DPIs).

*Sector*	Criteria													
	Resource yield	Distance to major roads	Distance to railway or port	Electricity accessibility	Distance to electrical grid	Distance to urban areas	Landcover	Inverse population density	Distance to oil and gas fields	Market accessibility	Land supply elasticity	Distance to demand centers	Distance to coal power plants	Active coal mine density
CSP	[0.309–0.542]	[0.068–0.209]	[0.022–0.045]	NA	[0.133–0.365]	[0.031–0.095]	[0.058–0.201]	NA	NA	NA	NA	NA	NA	NA
PV	[0.303–0.533]	[0.103–0.208]	[0.022–0.038]	NA	[0.113–0.354]	[0.044–0.139]	[0.060–0.223]	NA	NA	NA	NA	NA	NA	NA
Wind	[0.295–0.503]	[0.054–0.195]	[0.054–0.195]	NA	[0.130–0.342]	[0.018–0.037]	[0.038–0.111]	[0.025–0.082]	NA	NA	NA	NA	NA	NA
Hydro	[0.311–0.535]	[0.029–0.093]	[0.029–0.093]	NA	[0.060–0.167]	[0.022–0.044]	NA	[0.226–0.417]	NA	NA	NA	NA	NA	NA
Coal	[0.198–0.460]	[0.070–0.158]	[0.095–0.281]	[0.026–0.061]	NA	NA	NA	NA	NA	NA	NA	NA	[0.020–0.034]	[0.198–0.460]
CO	[0.355–0.597]	[0.031–0.084]	[0.041–0.116]	[0.063–0.211]	NA	NA	NA	NA	[0.152–0.393]	NA	NA	NA	NA	NA
CG	[0.288–0.555]	[0.055–0.137]	[0.026–0.045]	[0.078–0.372]	NA	NA	NA	NA	[0.173–0.456]	NA	NA	NA	NA	NA
UO	[0.281–0.575]	[0.102–0.362]	[0.031–0.073]	[0.049–0.171]	NA	NA	NA	NA	[0.102–0.362]	NA	NA	NA	NA	NA
UG	[0.253–0.555]	[0.101–0.365]	[0.030–0.060]	[0.049–0.170]	NA	NA	NA	NA	[0.120–0.423]	NA	NA	NA	NA	NA
MM	[0.300–0.635]	[0.095–0.390]	[0.095–0.390]	[0.052–0.167]	NA	NA	NA	NA	NA	NA	NA	NA	NA	NA
NMM	[0.241–0.613]	[0.065–0.287]	[0.065–0.287]	NA	NA	NA	NA	NA	NA	NA	NA	[0.140–0.449]	NA	NA
Crop	[0.196–0.661]	NA	NA	NA	NA	NA	NA	NA	NA	[0.143–0.571]	[0.108–0.493]	NA	NA	NA
Bio	[0.196–0.661]	NA	NA	NA	NA	NA	NA	NA	NA	[0.143–0.571]	[0.108–0.493]	NA	NA	NA

Weight ranges were derived from increasing and decreasing importance values by two points across all other criteria in the sector-specific AHP judgement matrix. Cell values of “NA” indicate criteria were not used for that DPI analysis and therefore not applicable for the uncertainty analysis too. Abbreviations of sector are as follows: CSP – concentrated solar power, PV – photovoltaic solar power, Wind – wind power, Hydro – hydropower, CO – conventional oil, CG – conventional gas, UO – unconventional oil, UG - unconventional gas, MM – metallic minerals, NMM – nonmetallic minerals, Crop – cropland expansion, Bio – biofuels expansion.

**Online-only Table 6:** Sensitivity analysis results summarized as average percentage change in bin cell counts.

*Sector*	Criteria													
	Resource yield	Distance to major roads	Distance to railway or port	Electricity accessibility	Distance to electrical grid	Distance to urban areas	Landcover	Inverse population density	Distance to oil and gas fields	Market accessibility	Land supply elasticity	Distance to demand centers	Distance to coal power plants	Active coal mine density
CSP	3, 10, 3, 2, 2	1, 4, 1, 3, 4	<1, 1, <1,< 1, 1	NA	1, 6, 2, 1, 8	1, 2, <1, 1, 5	1, 1, 1, 2, 2	NA	NA	NA	NA	NA	NA	NA
PV	2, 9, 3, 2, 4	2, 5, 1, 2, 4	<1, <1, <1, <1, 1	NA	2, 4, 1, 1, 7	1, 3, 1, 1, 6	1, 3, <1, 1, 5	NA	NA	NA	NA	NA	NA	NA
Wind	16, 18, 11, 6, 22	14, 5, 2, 2, 3	3, 1, 2, <1, 5	NA	31, 12, 7, 3, 10	2, 1, <1, <1, 2	18, 3, 1, 1, 1	19, 3, 1, <1, <1	NA	NA	NA	NA	NA	NA
Hydro	22, 17, 3, 30, 4	2, 1, <1, <1, 2	9, 1, <1, 2, 1	NA	10, 1, 1, 3, 1	1, 1, <1, 1, 2	NA	15, 15, 3, 18, 9	NA	NA	NA	NA	NA	NA
Coal	16, 2, 1, 19, 2	1, 2, <1, 10, 2	7, 1, 5, 13, 4	1, 1, <1, 5, 1	NA	NA	NA	NA	NA	NA	NA	NA	3, <1, <1, 3, 1	14, 5, 3, 46, 12
CO	28, 5, 8, 3, 10	1, 1, <1, 1	3, 1, 2, 2, <1	3, 4, 2, 1, 2	NA	NA	NA	NA	20, 2, 9, 1, 4	NA	NA	NA	NA	NA
CG	70, 14, 7, 13, 5	10, 2, <1, 2, <1	4, 1, <1, <1, 1	18, 3, <1, 1, 1	NA	NA	NA	NA	43, 14, 6, 12, 4	NA	NA	NA	NA	NA
UO	4, 28, 10, 3, 9	10, 5, 2, 3, 2	1, 2, 1, <1, 1	1, 1, 2, <1, 1	NA	NA	NA	NA	4, 6, 5, 1, 4	NA	NA	NA	NA	NA
UG	8, 10, 6, 7, 10	2, 2, 2, 1, 2	<1, <1, <1, <1, 1	2, <1, 1, <1, 1	NA	NA	NA	NA	3, 4, 1, 5, 3	NA	NA	NA	NA	NA
MM	2, 11, 5, 14, 13	1, 5, <1, 5, 4	3, 1, 3, 1, 1	1, 1, <1, 2, 1	NA	NA	NA	NA	NA	NA	NA	NA	NA	NA
NMM	3, 7, 14, 8, 15	8, 1, 1, 2, 3	1, <1, 2, 1, 2	NA	NA	NA	NA	NA	NA	NA	NA	6, 2, 6, 2, 6	NA	NA
Crop	57, 1, 2, 4, <1	NA	NA	NA	NA	NA	NA	NA	NA	36, 39, 9, 4, 3	70, 33, 6, 4, 1	NA	NA	NA
Bio	116, 8, 3, 14, 10	NA	NA	NA	NA	NA	NA	NA	NA	9, 18, 9, 4, 4	133, 19, 5, 6, 2	NA	NA	NA

Average percentages for each DPI value bins appear for each sector and criterion in the following order: 1 (>0.0–0.2), 2 (>0.2–0.4), 3 (>0.4–0.6), 4 (>0.6–0.8), and 5 (>0.8–1.0). Sensitivity analyses included 21 simulations, varying AHP weights from −20% to +20% of the original weight by increments of 2%. See individual DPI zip files^34^ for complete summary table generated from each simulation runs. Abbreviations of sector are as follows: CSP – concentrated solar power, PV – photovoltaic solar power, Wind – wind power, Hydro – hydropower, CO – conventional oil, CG – conventional gas, UO – unconventional oil, UG - unconventional gas, MM – metallic minerals, NMM – nonmetallic minerals, Crop – cropland expansion, Bio – biofuels expansion.
