# Supplementary material for: Mapping global development potential for renewable energy, fossil fuels, mining and agriculture sectors
Source: Sci Data. 2019 Jun 27;6:101. doi: 10.1038/s41597-019-0084-8 (PMC6597728; doi:10.1038/s41597-019-0084-8)
Supplement: Supplementary file 2 — Supplementary Information [file 41597_2019_84_MOESM2_ESM.pdf]

Supplementary Information

Mapping global development potential for renewable energy, fossil fuels, mining and agriculture sectors

James R. Oakleaf[1*†], Christina M. Kennedy[1†], Sharon Baruch-Mordo[1], James S. Gerber[2], Paul C. West[2], Justin A. Johnson[3] and Joseph Kiesecker[1]

[*]Corresponding author joakleaf@tnc.org

[†]Authors contributed equally.

Table of Contents *Pages*

Supplementary Table S1. Final classified development potential index (DPI) map per sector with corresponding Analytic Hierarchy Process (AHP) and Weighted Linear Combination (WLC) inputs………………………………. 2 – 14

Supplementary Fig. S1. Example sensitivity results for minimum and maximum criteria weight changes applied to the active coal mining density (ACDM) criteria within the coal development potential index (DPI) analysis……………………………………………………………………………...18 – 19

Supplementary Table S1. Final classified development potential index (DPI) map per sector with corresponding Analytic Hierarchy Process (AHP) and Weighted Linear Combination (WLC) inputs.

Sector	AHP/WLC Methods and Output (step 3)
Concentrated Solar Power (CSP)	*Classified DPI Map*

Classified DPI Map

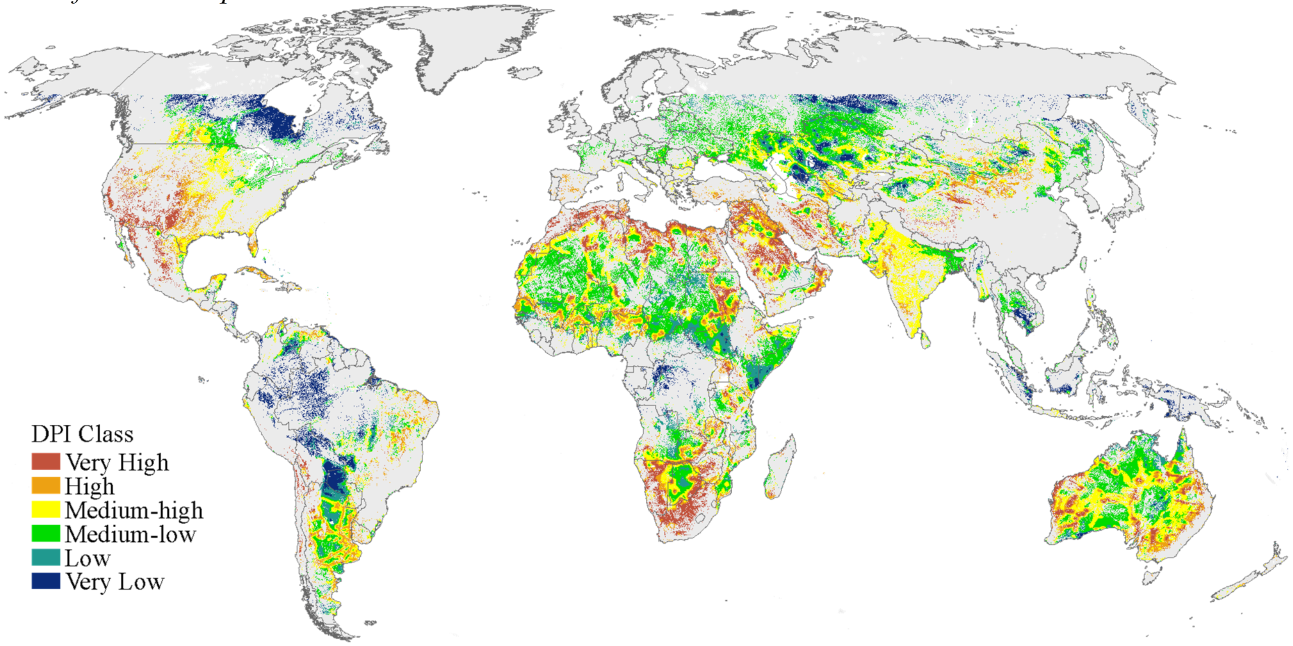

AHP pairwise comparison values:

Criteria	Resource yield	Distance to electrical grid	Distance to major roads	Landcover	Distance to urban areas	Distance to railways or ports
RY	1	3	5	5	7	7
DEG	1/3	1	3	3	5	7
DMR	1/5	1/3	1	1	3	7
Landcover	1/5	1/3	1	1	3	5
DUA	1/7	1/5	1/3	1/3	1	3
DRP	1/7	1/7	1/7	1/5	1/3	1
Criteria weights:	0.451	0.241	0.119	0.109	0.051	0.029

Notes on comparison values: Similar other solar suitability assessments[1,2], we ranked resource yield (RY) as the most important criterion, followed closely in importance by distance to the electrical grid (DEG), and then distance to major roads (DMR). Following ref[2], we ranked landcover as equal in influence with DMR. Distance to urban areas (DUA) was ranked as the lowest importance of all other criteria expect distance to railways and ports (DRP) based on ref[1]. Given that ref[3] identified distance to railroads as influential for siting of solar power and because both rail cars and ships provide an inexpensive option for transporting building materials for any renewable energy project, we included the DRP criteria but ranked it least important.

Photovoltaic Solar (PV) Power

Classified DPI Map

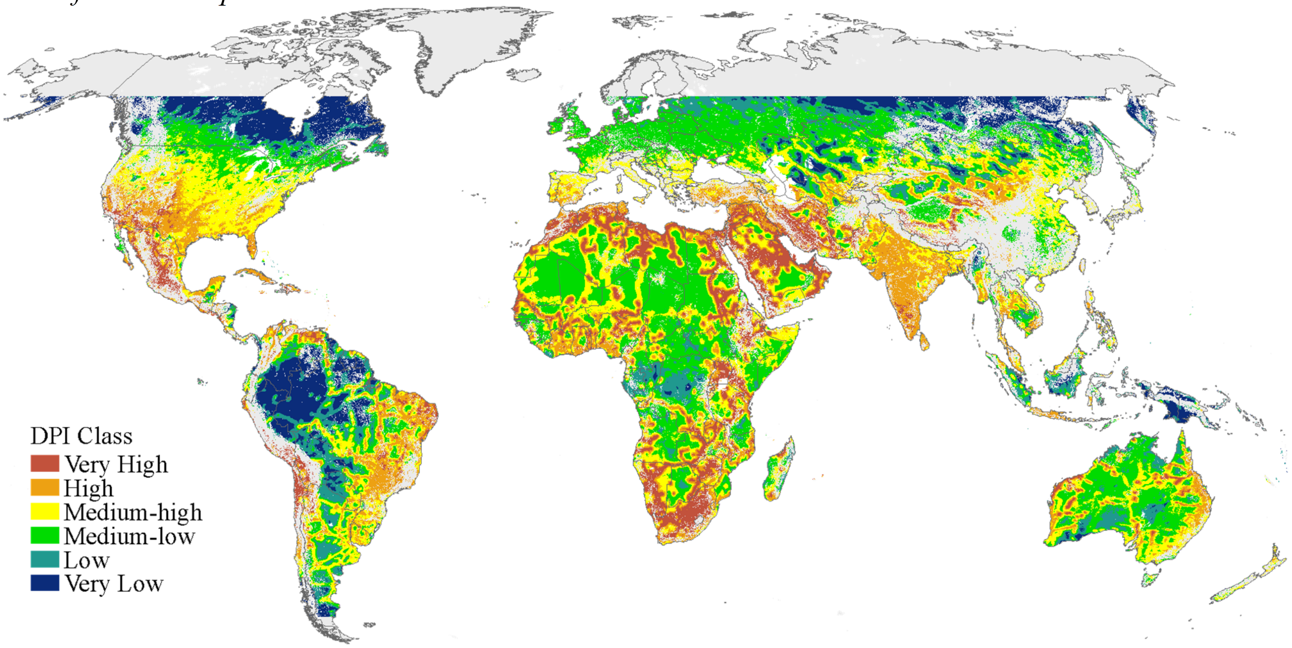

AHP pairwise comparison values:

Criteria	Resource yield	Distance to electrical grid	Distance to major roads	Landcover	Distance to urban areas	Distance to railways or ports
RY	1	3	5	5	5	9
DEG	1/3	1	3	3	3	7
DMR	1/5	1/3	1	1	3	5
Landcover	1/5	1/3	1	1	3	5
DUA	1/5	1/3	1/3	1/3	1	5
DRP	1/9	1/7	1/5	1/5	1/5	1
Criteria weights:	0.447	0.228	0.115	0.115	0.069	0.026

Notes on comparison values: Similar to CSP, we followed other solar suitability assessments[1,2] and ranked resource yield (RY) as the most important criterion, followed closely in importance by distance to the electrical grid (DEG), and then distance to major roads (DMR). Following ref[2], we ranked landcover as equal in influence with DMR. Distance to urban areas (DUA) was ranked as the lowest importance of all other criteria except distance to railways and ports (DRP) based on ref[1]; however, we increased the importance value when comparing to CSP due to PV installations being more commonly located in or near developed areas[4]. Given that ref[3] identified distance to railroads as influential for siting of solar power and because both rail cars and ships provide an inexpensive option for transporting building materials for any renewable energy project, we included the DRP criteria but ranked it least important. This criterion was given an even lower comparison values than CSP, because less materials are required for PV development[5].

Wind Power

Classified DPI Map

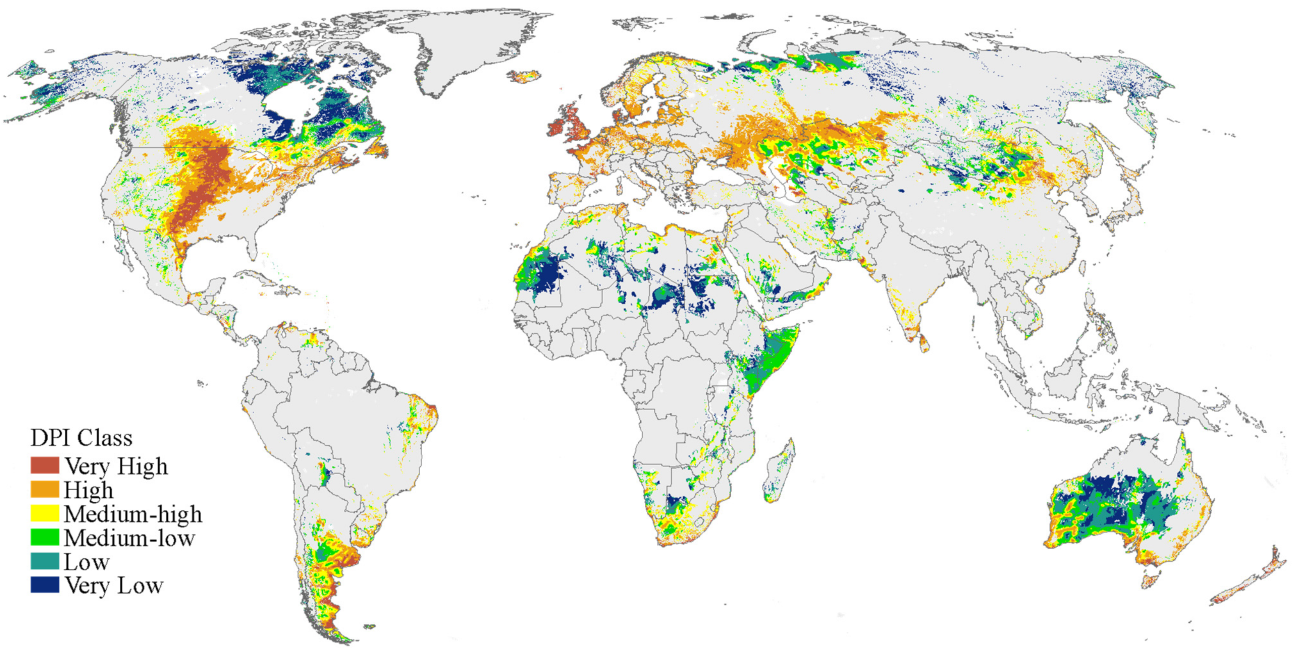

AHP pairwise comparison values:

Criteria	Resource yield	Distance to electrical grid	Distance to railways or ports	Distance to major roads	Landcover	Inverse population density	Distance to urban areas
RY	1	3	5	5	7	7	9
DEG	1/3	1	3	3	5	5	7
DRP	1/5	1/3	1	1	3	3	5
DMR	1/5	1/3	1	1	3	3	5
Landcover	1/7	1/5	1/3	1/3	1	3	5
IPD	1/7	1/5	1/3	1/3	1/3	1	3
DUA	1/9	1/7	1/5	1/5	1/5	1/3	1
Criteria weights:	0.422	0.23	0.109	0.109	0.065	0.042	0.023

Notes on comparison values: Similar to other wind suitability studies[2,6,7], we ranked resource yield (RY) as the most important criterion, followed closely in importance by distance to the electrical grid (DEG). Given the need to transport oversized wind development materials (i.e., hubs and blades) and a likelihood of these sizes continuing to increase[8], we assigned the distance to railways and ports (DRP) and the distance to major roads (DRM) criteria as equally important and more important than landcover, inverse population density (IPD), and distance to urban areas (DUA). We ranked the landcover criterion as more important than both IDP and DUA because of a greater frequency of use in other wind suitability studies[2,6,7]. Finally, because of a trend towards larger power producing wind farms and longer transmission distances[9,10], we graded the IPD criterion as more important than DUA.

Hydropower

Classified DPI Map

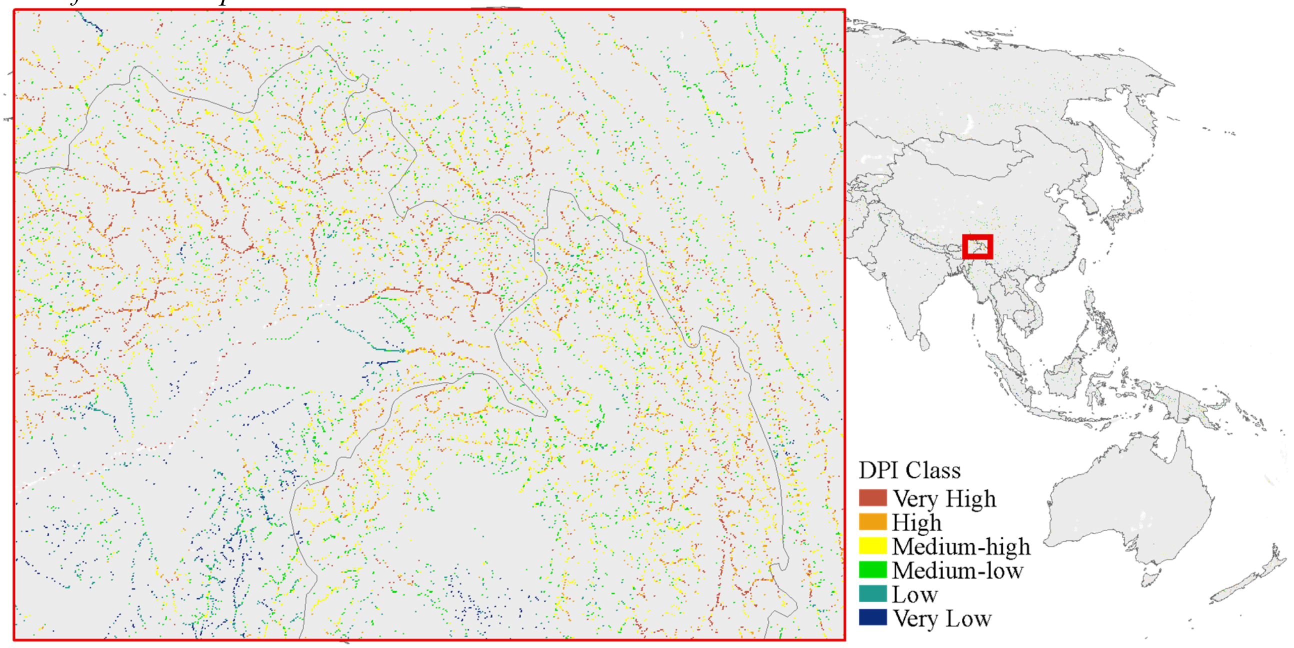

AHP pairwise comparison values:

Criteria	Resource yield	Inverse population density	Distance to electrical grid	Distance to major roads	Distance to railways or ports	Distance to urban areas
RY	1	3	5	7	7	9
IPD	1/3	1	5	7	7	9
DEG	1/5	1/5	1	3	3	5
DMR	1/7	1/7	1/3	1	1	3
DRP	1/7	1/7	1/3	1	1	3
DUA	1/9	1/9	1/5	1/3	1/3	1
Criteria weights:	0.453	0.309	0.11	0.051	0.051	0.026

Notes on comparison values: We ranked resource yield (RY) as more important than all other criteria, because of the substantial cost benefits associated with building a hydropower dam in regions with high resource potential[11]. Given that studies identify population displacement as a substantial hurdle when establishing a reservoir[12], we assigned the inverse population density (IPD) criterion to be slightly less important than RY but more important than all other criteria. When comparing the distance to the electrical grid (DEG) criteria with either distance to major roads (DMR) or distance to railways or ports (DRP), we ranked DEG more important than these two criteria based on ref[13], which ranked grid connection as a higher risk on investment for building a hydropower plant than any transportation criteria (i.e., DMR or DRP). We assigned equal importance when comparing DMR and DRP criteria because materials for development can occur from either. Similar to the wind MCDA, we weighted distance to urban areas (DUA) as least important, because large transmission distances are typical for large hydropower dams (i.e., > 30 MW; ref[14]), which is a size most common for future hydropower projects[15].

Coal Mining

Classified DPI Map

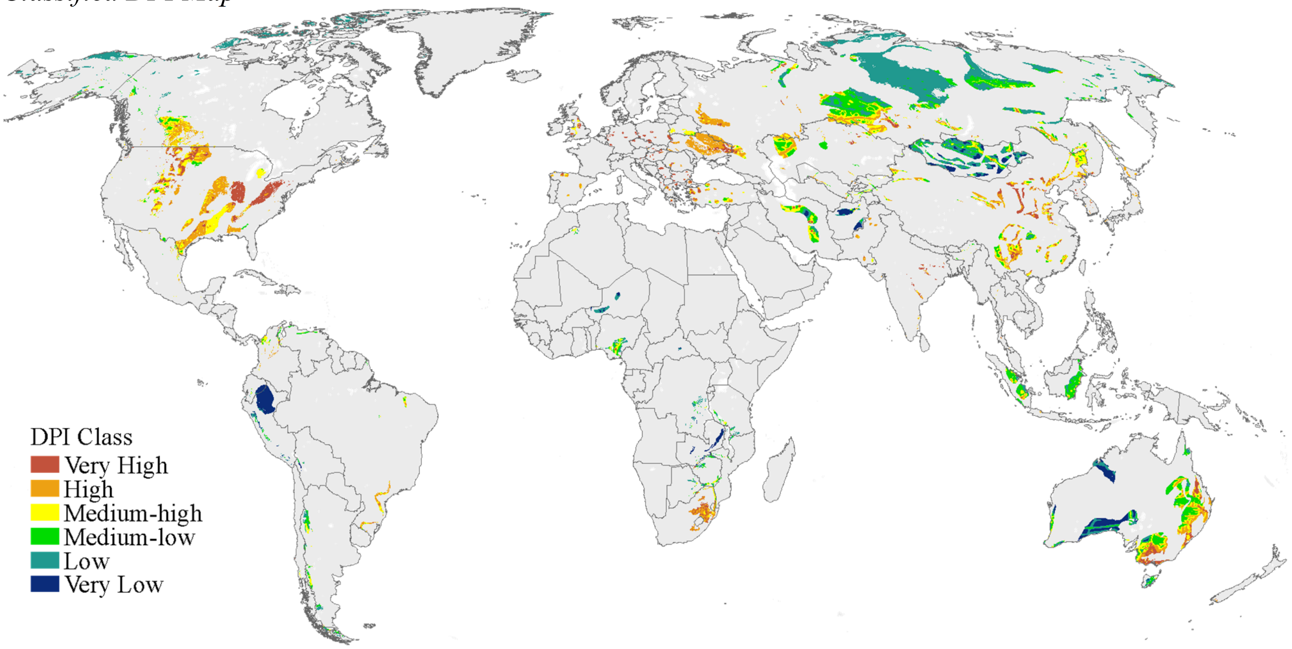

AHP pairwise comparison values:

Criteria	Resource yield	Active coal mine density	Distance to railways or ports	Distance to major roads	Electricity accessibility	Distance to coal power plants
RY	1	1	3	5	7	9
ACMD	1	1	3	5	7	9
DRP	1/3	1/3	1	3	5	7
DMR	1/5	1/5	1/3	1	5	7
EA	1/7	1/7	1/5	1/5	1	3
DCPP	1/9	1/9	1/7	1/7	1/3	1
Criteria weights:	0.336	0.336	0.164	0.101	0.04	0.023

Notes on comparison values: We assigned resource yield (RY) and active coal mine density (ACMD) criteria to be equally important and higher than all other criteria, because existing coal mines occur within a coal basin where it is most profitable to operate due to factors such as low overburden, large coal seams, and/or high-quality coal[16]; and future development will seek to expand and exhaust currently mined deposits before moving to unproven deposits[17]. After RY and ACMD, we considered distance to railway and ports (DRP) and distance to major roads (DMR) criteria as the next most important given that coal transportation is a critical element for coal field development[17]. We ranked DRP slightly higher than DMR given that 70% of U.S. coal is transported by either rail or ships[18] , however, in developing countries there is still a strong dependence on trucking coal[19]. After transportation, we considered access to electricity (AE) more important than the distance from coal-fired power plants (DCPP) because of the large benefit electricity provides for all types of mining[20] and the large variation in distances existing power plants are from their coal source[17].

Conventional Oil

Classified DPI Map

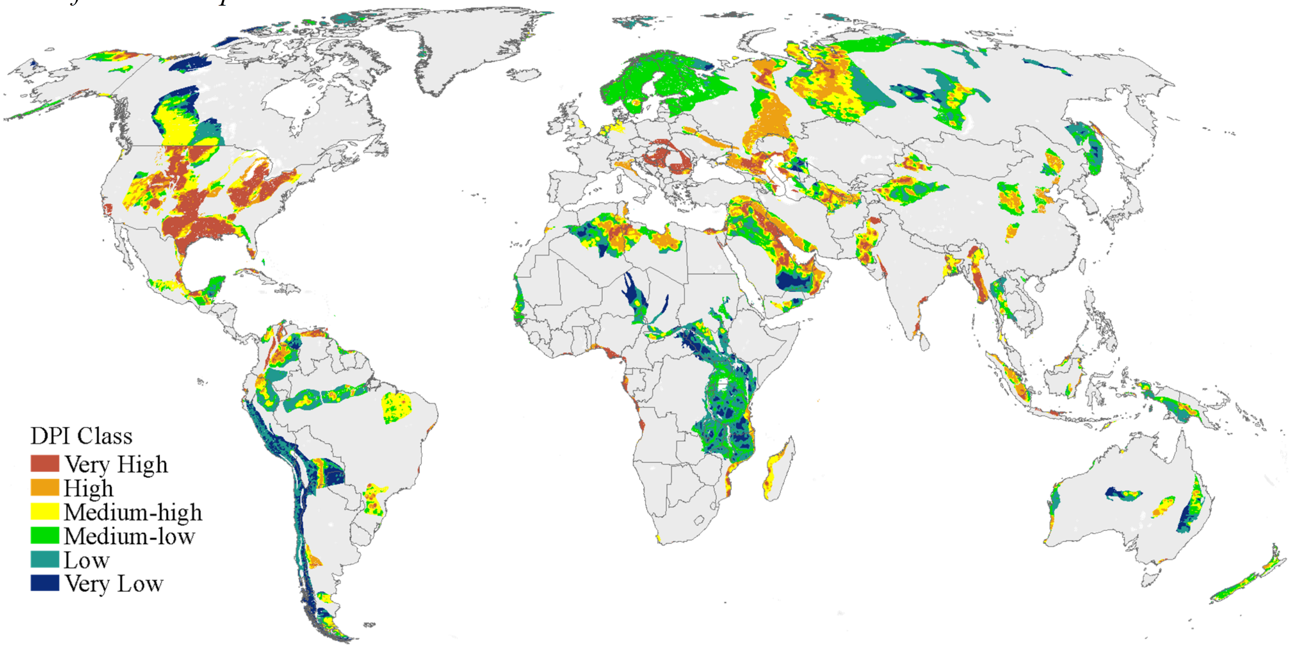

AHP pairwise comparison values:

Criteria	Resource yield	Distance to producing oil/gas fields	Electricity accessibility	Distance to railways or ports	Distance to major roads
RY	1	3	5	7	7
DPOG	1/3	1	3	5	5
EA	1/5	1/3	1	3	3
DRP	1/7	1/5	1/3	1	3
DMR	1/7	1/5	1/3	1/3	1
Criteria weights:	0.507	0.257	0.123	0.069	0.044

Notes on comparison values: We ranked resource yield (RY) as the most important criterion, because fossil fuel development often occurs in remote places with high untapped resources[21,22]. After yield, we ranked distance to producing oil/gas fields (DPOGF) as the second most important criteria given the higher likelihood of infilling and expansion of existing oil and gas fields relative to the development of new basins that require much higher capital[23]. Because oil development relies on electricity to pump and collect resources[24], we ranked access to electricity (EA) as more important than either distance to railways or ports (DRP) or distance to major roads (DMR). Finally given the high dependency on rail and ship transportation for oil resources[24], we ranked DRP more important than DMR but only slightly due to heavy road use in the all phases of oil field development[21,25].

Conventional Gas

Classified DPI Map

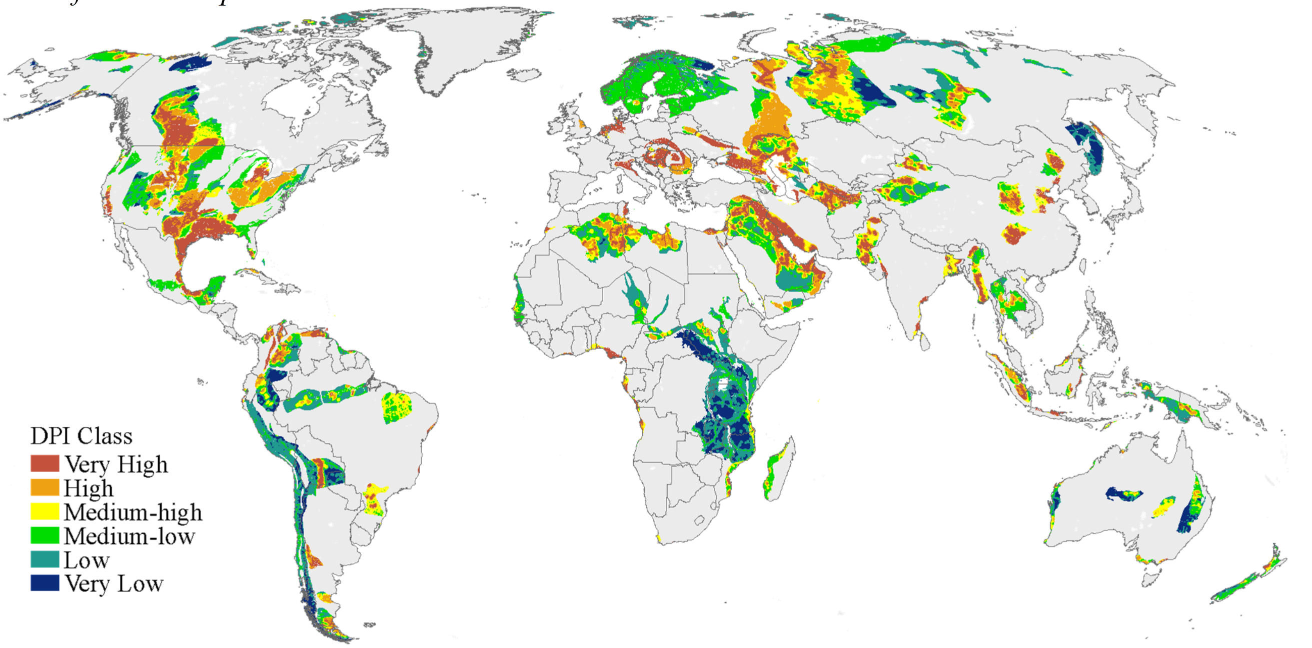

AHP pairwise comparison values:

Criteria	Resource yield	Distance to producing oil/gas fields	Electricity accessibility	Distance to major roads	Distance to railways or ports
RY	1	2	5	5	9
DPOG	1/2	1	3	5	7
EA	1/5	1/3	1	3	5
DMR	1/5	1/5	1/3	1	5
DRP	1/9	1/7	1/5	1/5	1
Criteria weights:	0.455	0.294	0.137	0.082	0.032

Notes on comparison values: We ranked resource yield (RY) as the most important criterion, because fossil fuel development often occurs in remote places with high untapped resources[21,22]. After yield, we ranked distance to producing oil/gas fields (DPOGF) as the second most important criteria given the higher likelihood of infilling and expansion of existing oil and gas fields relative to the development of new basins that require much higher capital[23]. We assigned DPOGF as slightly higher importance value than applied in the conventional oil MCDA due to higher infrastructure needs being required to fully capture gas[24]. Because gas development relies on electricity to pump and collect resources[24], we ranked access to electricity (AE) as more important than either distance to railways or ports (DRP) or distance to major roads (DMR). We ranked DMR more important than DRP because of heavy road use in the all phases of gas field development[21,25] and the transportation of natural gas via ships and rail limited to extremely costly liquid natural gas (LNG)[26]. We did not however remove the DRP criterion due to an increase in LNG production and exports for large gas producing countries like the US[27].

Unconventional Oil

Classified DPI Map

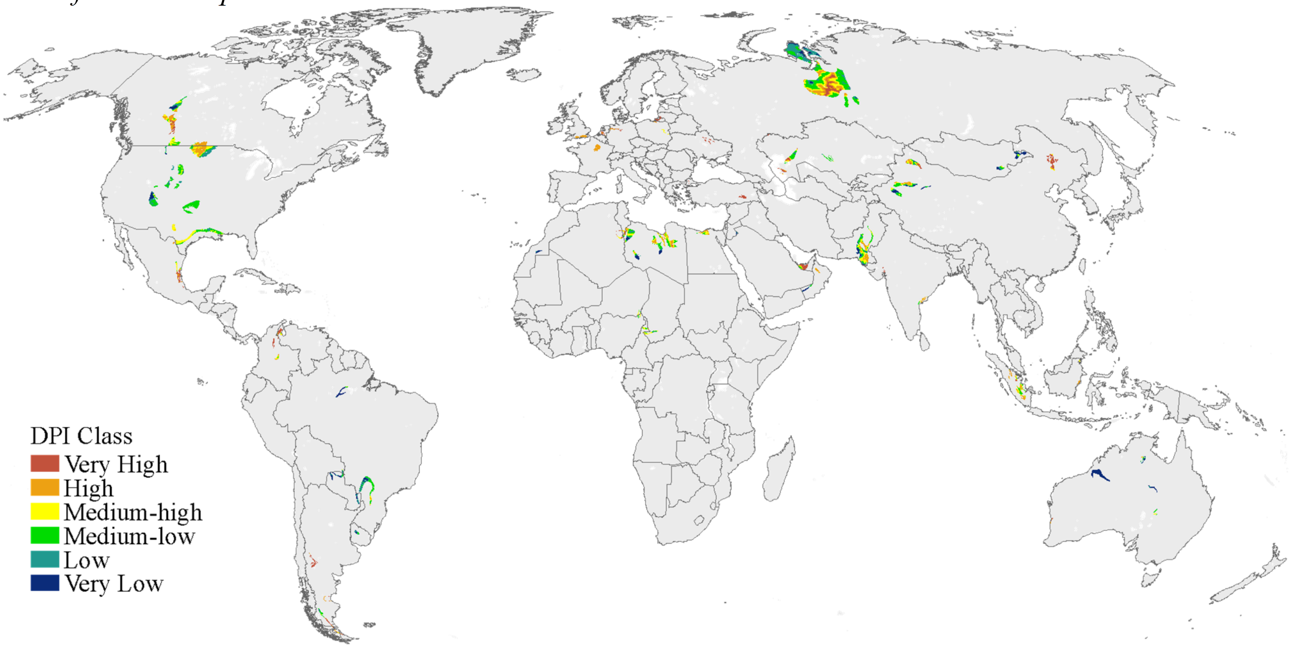

AHP pairwise comparison values:

Criteria	Resource yield	Distance to producing oil/gas fields	Distance to major roads	Electricity accessibility	Distance to railways or ports
RY	1	3	3	5	7
DPOGF	1/3	1	1	3	5
DMR	1/3	1	1	3	5
EA	1/5	1/3	1/3	1	3
DRP	1/7	1/5	1/5	1/3	1
Criteria weights:	0.469	0.201	0.201	0.086	0.043

Notes on comparison values: We ranked resource yield (RY) as the most important criterion, because fossil fuel development often occurs in remote places with high untapped resources[21,22]. After yield, we ranked distance to producing oil/gas fields (DPOGF) as the second most important criteria given the higher likelihood of infilling and expansion of existing oil and gas fields relative to the development of new basins that require much higher capital[23]. Because fracking liquids used in unconventional oil practices are almost exclusively transported by trucks[28], we considered distance to major roads (DMR) as more important than both electricity accessibility (EA) or distance to railways or ports (DRP). EA was given a slightly higher ranking than DRP because oil development relies on electricity to pump and collect resources[24]. Finally given the high dependency on rail and ship transportation for oil resources[24], we included DRP but ranked it least important.

Unconventional Gas

Classified DPI Map

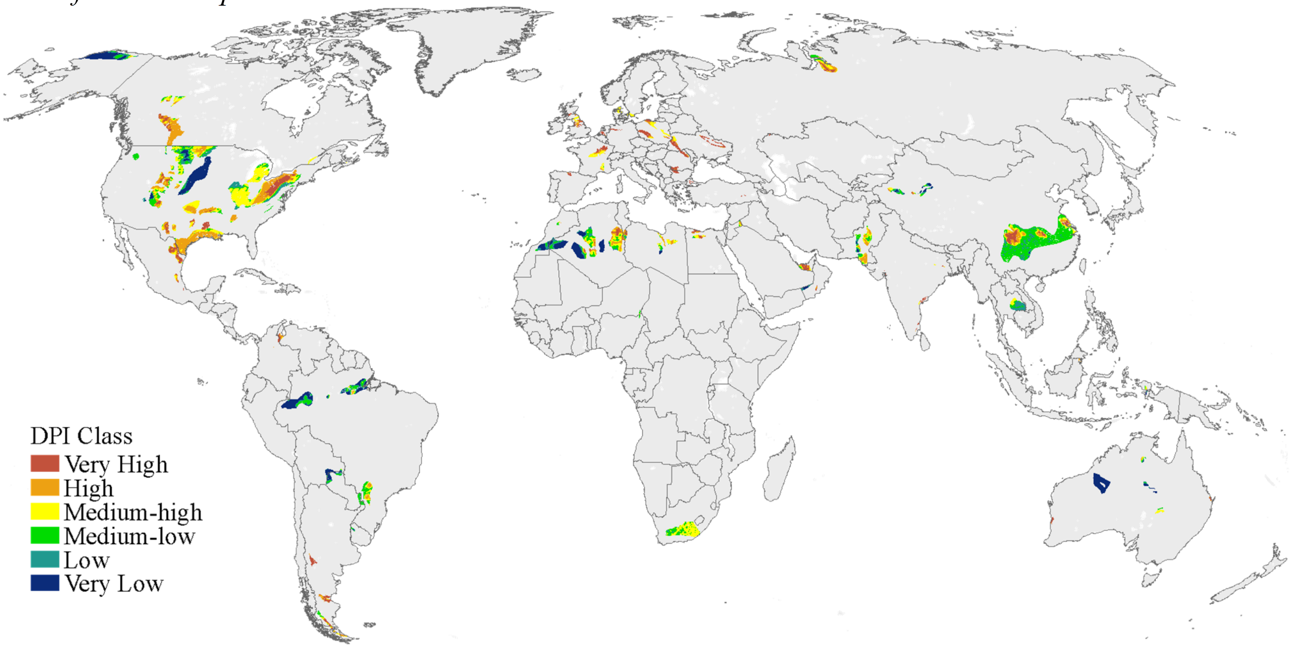

AHP pairwise comparison values:

Criteria	Resource yield	Distance to producing oil/gas fields	Distance to major roads	Electricity accessibility	Distance to railways or ports
RY	1	2	3	5	9
DPOGF	1/2	1	1	3	7
DMR	1/3	1	1	3	5
EA	1/5	1/3	1/3	1	3
DRP	1/9	1/7	1/5	1/3	1
Criteria weights:	0.447	0.23	0.201	0.085	0.037

Notes on comparison values: We ranked resource yield (RY) as the most important criterion, because fossil fuel development often occurs in remote places with high untapped resources[21,22]. After yield, we ranked distance to producing oil/gas fields (DPOGF) as the second most important criteria given the higher likelihood of infilling and expansion of existing oil and gas fields relative to the development of new basins that require much higher capital[23]. We assigned DPOGF as slightly higher importance value than applied in the unconventional oil MCDA due to higher infrastructure needs being required to fully capture gas[24]. Because fracking liquids used in unconventional gas practices are almost exclusively transported by trucks[28], we considered distance to major roads (DMR) as more important than both electricity accessibility (EA) or distance to railways or ports (DRP). EA was given a slightly higher ranking than DRP because gas development relies on electricity to pump and collect resources[24]. Finally given the limited current role of rail and ship transportation for gas resources[26], we ranked DRP least important but still included this criterion due to this transport method having a potential to increase in the future[27].

Metallic Mining

Classified DPI Map

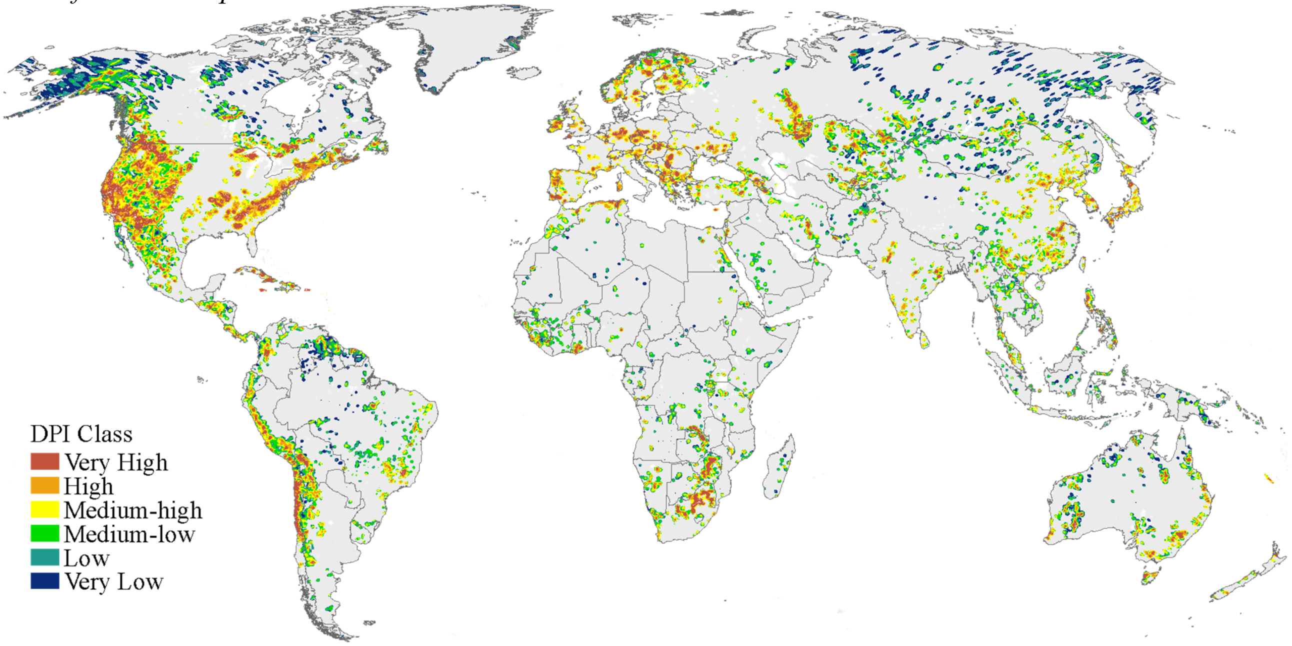

AHP pairwise comparison values:

Criteria	Resource yield	Distance to major roads	Distance to railways or ports	Electricity accessibility
RY	1	3	3	5
DMR	1/3	1	1	3
DRP	1/3	1	1	3
EA	1/5	1/3	1/3	1
Criteria weights:	0.522	0.200	0.200	0.078

Notes on comparison values: Because sufficient sized mineral deposits are critical for any mining development[29], we ranked resource yield (RY) as the most important criterion. With proximity to transportation infrastructure being the next prerequisite for mining to occur[29,30], we considered distance to major roads (DMR) and distance to railways and ports (DRP) only moderately less important than resource yield. In comparing these two criteria to each other, we ranked them equally important due to transportation preference often being related to the value and size of mineral being mined[31] and, thus, hard to distinguish within our MCDA. Finally, we also included electricity accessibility (EA) as a feasibility criterion due to the vast amount of energy commonly used to mine and process metallic minerals at the mine site[32], but ranked EA as the least important criteria due to on-site energy production often occurring in remote mining locations [31,33].

Non-metallic Mining

Classified DPI Map

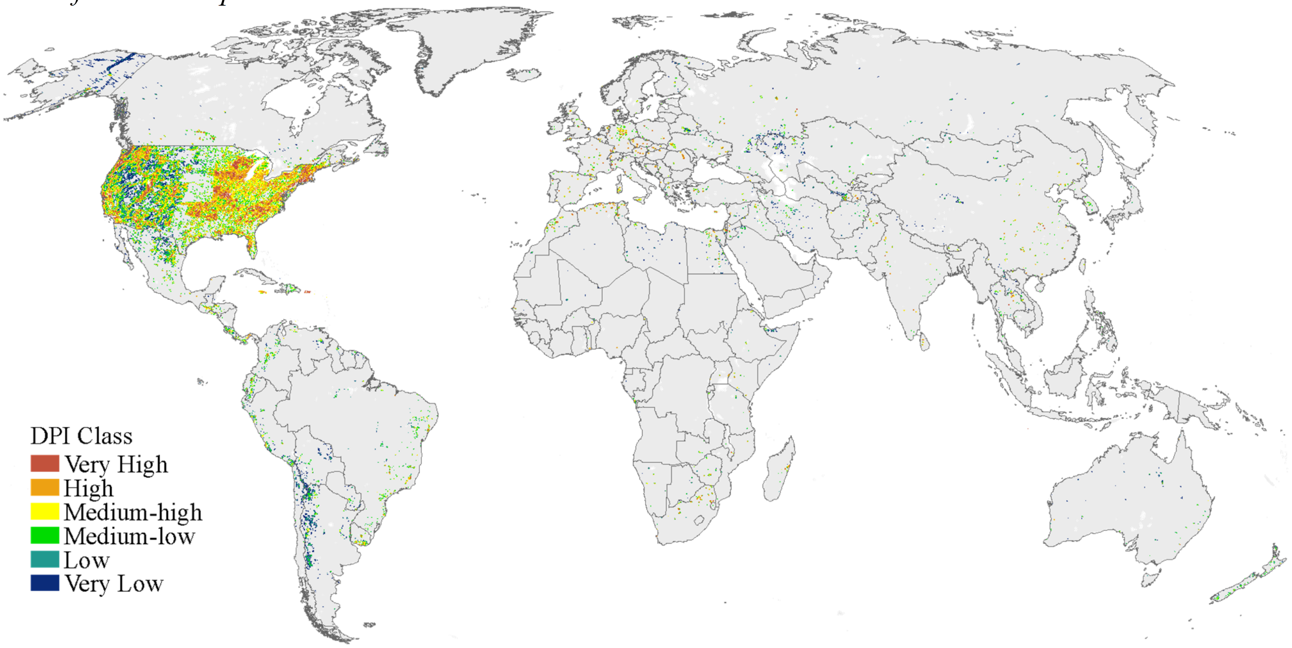

AHP pairwise comparison values:

Criteria	Resource yield	Distance to aggregate demand centers	Distance to major roads	Distance to railways or ports
RY	1	3	3	3
DADC	1/3	1	3	3
DMR	1/3	1/3	1	1
DRP	1/3	1/3	1	1
Criteria weights:	0.488	0.276	0.118	0.118

Notes on comparison values: Because sufficient sized mineral deposits are critical for any mining development[29], we ranked resource yield (RY) as the most important criterion. With non-metallic mining being heavily dependent on proximity to populated areas that use the materials mined[34–36], we assigned distance to aggregate demand centers (DADC) as more important than either distance to major roads (DMR) or distance to railways or ports (DRP). Finally, we ranked DMR and DRP equally since all forms of transportation (i.e., truck, train, and ship) are commonly used and transportation selection is based on whichever type is closest to the mine site and can move materials to the demand center[37].

Croplands Expansion

Classified DPI Map

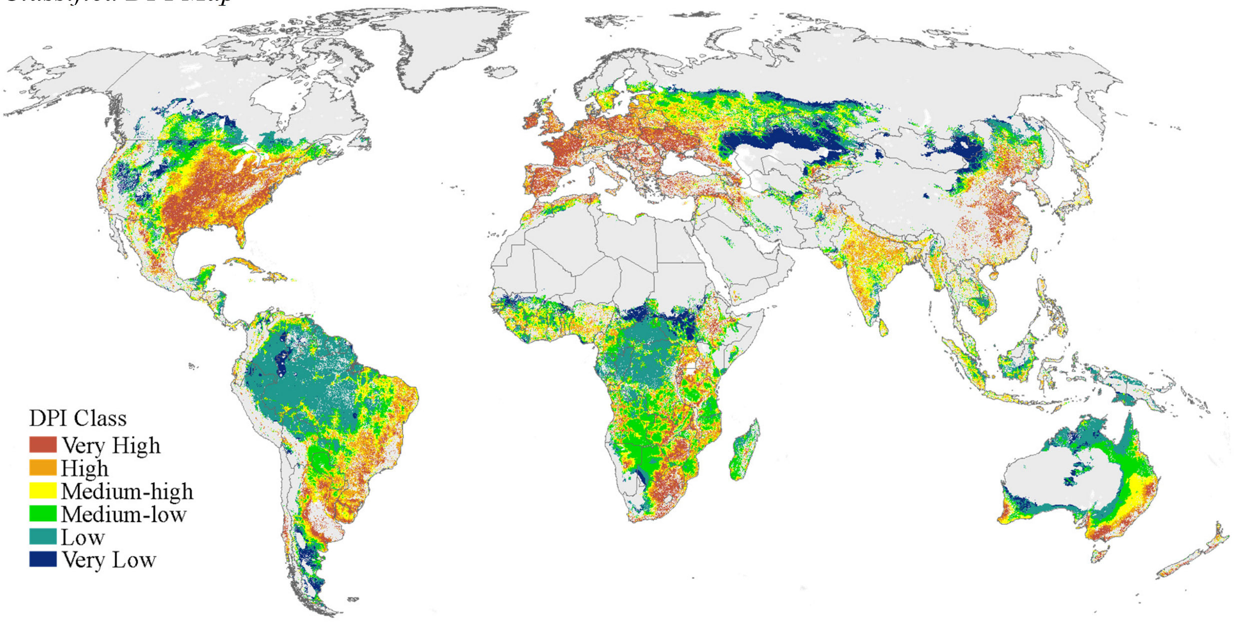

AHP pairwise comparison values:

Criteria	Resource yield	Market accessibility	Land supply elasticity
Resource yield	1	2	2
Market accessibility	1/2	1	2
Land supply elasticity	1/2	1/2	1
Criteria weights:	0.493	0.311	0.196

Notes on comparison values: In addition to resource yield (RY) criteria, we included two feasibility criteria within our cropland expansion MCDA: market accessibility (MA) as proxied by travel time to cities[38] and land supply elasticity (LSE) map as proxied by nighttime lights[39]. Next to RY, market access is a widely recognized contributor to cropland expansion in both developing and developed countries[40–42]. At the same time, lands close to major urban areas compete with more profitable land uses such as residential and built-up areas and thus exhibit a lower LSE[43,44] and have a higher likelihood of crop intensification than crop expansion[45]. Therefore, in our AHP comparisons, we ranked RY only slightly more important than both MA and LSE; and ranked MA slightly more important than LSE, because MA is cited more often in the literature as a factor affecting cropland expansion[41].

Biofuels Expansion

Classified DPI Map

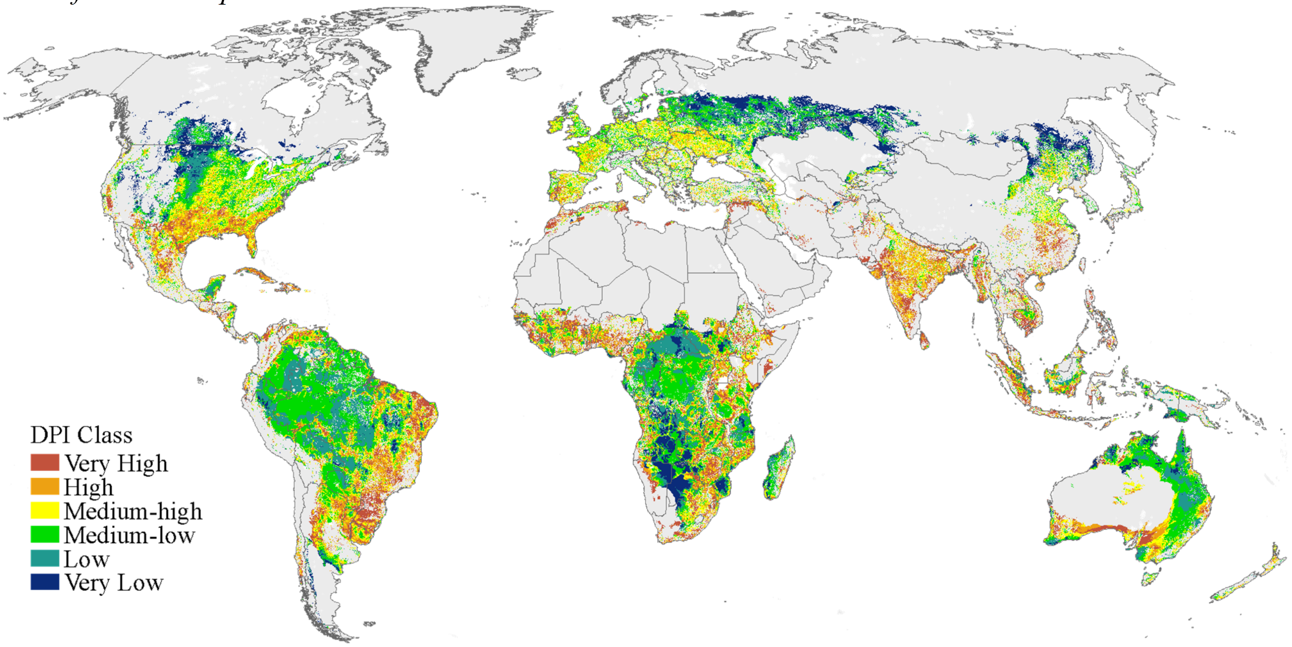

AHP pairwise comparison values:

Criteria	Resource yield	Market accessibility	Land supply elasticity
Resource yield	1	2	2
Market accessibility	1/2	1	2
Land supply elasticity	1/2	1/2	1
Criteria weights:	0.493	0.311	0.196

Notes on comparison values: Similar to cropland expansion, we included with our biofuels resource yield (RY) criterion, two feasibility criteria: market accessibility (MA) as proxied by travel time to cities[38] and land supply elasticity (LSE) map as proxied by nighttime lights[39]. We followed same logic for criteria comparisons as cropland expansion ranking RY only slightly more important than both MA and LSE; and ranked MS slightly more important than LSE.

Metallic mining development potential index (DPI) example: Methods applied in generating criteria weight ranges for DPI uncertainty analysis.

We provide a detailed example for Metallic Mining (MM) DPI of how we define criteria weights ranges. Ranges were iteratively sampled with Monte Carlo procedures described in main text Uncertainty section. This process was sector- and criterion-specific and was applied for all 13 DPIs and their criteria (see Online-only Table 5 for all criteria and sector weight range results).

For all criteria related to the MM DPI (Supplementary Table S2), we modified the importance value (i.e., 1-9 and reciprocals) of the original AHP judgement matrix (e.g., Supplementary Table S3). We increased scores two points up (Supplementary Tables S4, S6, S8, and S10) or down (Supplementary Tables S5, S7, S9, and S11), and for each score modification, we adjusted the criterion's judgement matrix column and row (highlighted in grey) to maintain consistency ratios within the < 0.1 guideline[46,47]. This process required two additional AHP matrices for each sector and criterion combination, which produced 8 new matrices for MM DPI given it had 4 criteria, and overall produced 130 new AHP matrices across all sectors and criteria.

Supplementary Table S2. Metallic mining ranges of criteria weights for uncertainty analysis. Summary of results from Tables S4 – S11.

Criteria	Lowest criteria weight with -2 importance value	High criteria weight with +2 importance value
Resource Yield (RY)	0.300	0.635
Distance to Major Roads (DMR)	0.095	0.390
Distance to Railways and Ports (DRP)	0.095	0.390
Electricity Access (EA)	0.052	0.167

Supplementary Table S3. Original Analytical Hierarchy Process (AHP) comparison table used for Metallic Mining (MM) Development Potential Index (DPI).

AHP pairwise comparison values:

Criteria	Resource Yield (RY)	Distance to Major Roads (DMR)	Distance to Railways or Ports (DRP)	Electricity Accessibility (EA)
RY	1	3	3	5
DMR	1/3	1	1	3
DRP	1/3	1	1	3
EA	1/5	1/3	1/3	1
Criteria weights:	0.552	0.200	0.200	0.078

Supplementary Table S4. RY +2 importance value changes. Shaded cells show modified importance values with bolded weight value indicating the resulting criteria weight used for range.

Criteria	Resource Yield (RY)	Distance to Major Roads (DMR)	Distance to Railways or Ports (DRP)	Electricity Accessibility (EA)
RY	1	5	5	7
DMR	1/5	1	1	3
DRP	1/5	1	1	3
EA	1/7	1/3	1/3	1
	**0.635**	0.151	0.151	0.063

Supplementary Table S5. RY -2 importance value changes. Same table description as Table S3.

RY	1	1	1	3
DMR	1	1	1	3
DRP	1	1	1	3
EA	1/3	1/3	1/3	1
	**0.300**	0.300	0.300	0.100

Supplementary Table S6. DMR +2 importance value changes. Same table description as Table S3.

RY	1	1	3	5
DMR	1	1	3	5
DRP	1/3	1/3	1	3
EA	1/5	1/5	1/3	1
	0.390	**0.390**	0.152	0.068

Supplementary Table S7. DMR -2 importance value changes. Same table description as Table S3.

RY	1	5	3	5
DMR	1/5	1	1/3	1
DRP	1/3	3	1	3
EA	1/5	1	1/3	1
	0.560	**0.095**	0.249	0.095

Supplementary Table S8. DRP +2 importance value changes. Same table description as Table S3.

Criteria	Resource Yield (RY)	Distance to Major Roads (DMR)	Distance to Railways or Ports (DRP)	Electricity Accessibility (EA)
RY	1	3	1	5
DMR	1/3	1	1/3	3
DRP	1	3	1	5
EA	1/5	1/3	1/3	1
	0.390	0.152	**0.390**	0.068

Supplementary Table S9. DRP -2 importance value changes. Same table description as Table S3.

RY	1	3	5	5
DMR	1/3	1	3	3
DRP	1/5	1/3	1	1
EA	1/5	1/3	1/3	1
	0.560	0.249	**0.095**	0.095

Supplementary Table S10. EA +2 importance value changes. Same table description as Table S3.

RY	1	3	3	3
DMR	1/3	1	1	1
DRP	1/3	1	1	1
EA	1/3	1	1	1
	0.500	0.167	0.167	**0.167**

Supplementary Table S11. EA -2 importance value changes. Same table description as Table S3.

RY	1	3	3	7
DMR	1/3	1	1	5
DRP	1/3	1	1	5
EA	1/7	1/5	1/5	1
	0.528	0.210	0.210	**0.052**

Supplementary Fig. S1. Example sensitivity results for minimum and maximum criteria weight changes applied to the active coal mining density (ACDM) criteria within the coal development potential index (DPI) analysis. Mapped examples of sensitivity analysis for the ACDM criterion within the Coal DPI comparing the minimum (i.e., -20%) and maximum (i.e., +20%) criterion weight change with the original coal DPI (DPI_{Orig}). Row (a) compares binned DPI maps and percentage of cells falling within each bin. Row (b) highlights the spatial difference in the continuous coal DPI maps with row (c) showing cell-by-cell continuous value histograms and the binned breakpoints associated with row (a). Finally, row (d) displays the final change maps for the minimum and maximum criterion weight change and highlights those cells within the greatest change from the DPI_{Orig}. This example is one of three criteria within DPI sectors (i.e., resource yield - wind, resource yield - hydropower, ACDM - coal) which had a more detailed sensitivity analysis ran to further examine absolute cell value changes as reported in Table 3.

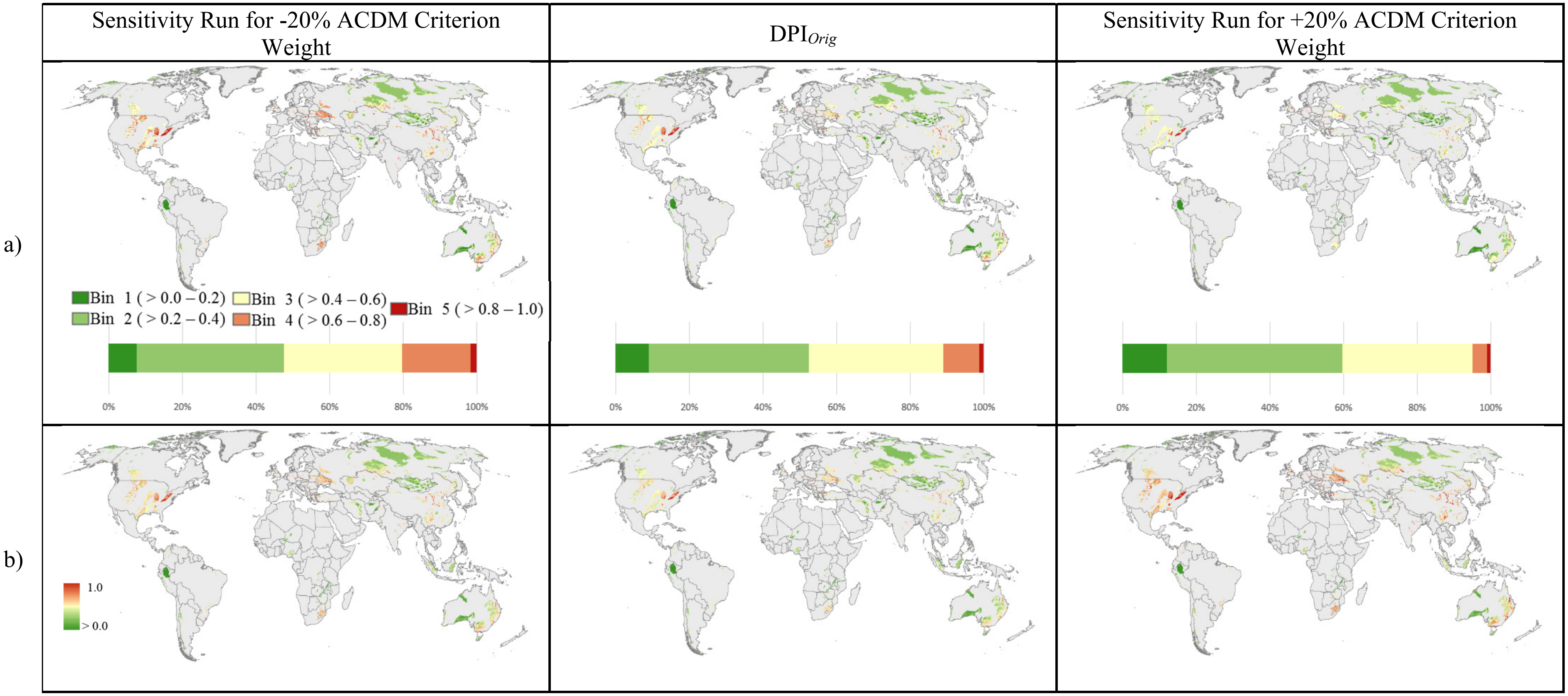

c)

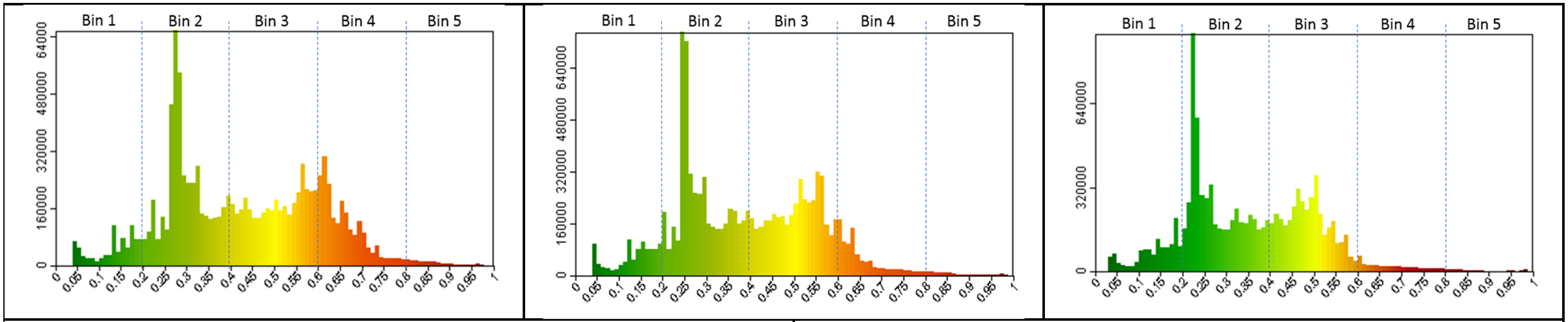

Bin 1
Bin 2
Bin 3
Bin 4
Bin 5
0
160000
320000
480000
640000
0
0.05
0.1
0.15
0.2
0.25
0.3
0.35
0.4
0.45
0.5
0.55
0.6
0.65
0.7
0.75
0.8
0.85
0.9
0.95
1
Bin 1
Bin 2
Bin 3
Bin 4
Bin 5
0
160000
320000
480000
640000
Bin 1
Bin 2
Bin 3
Bin 4
Bin 5
0
320000
640000

d)

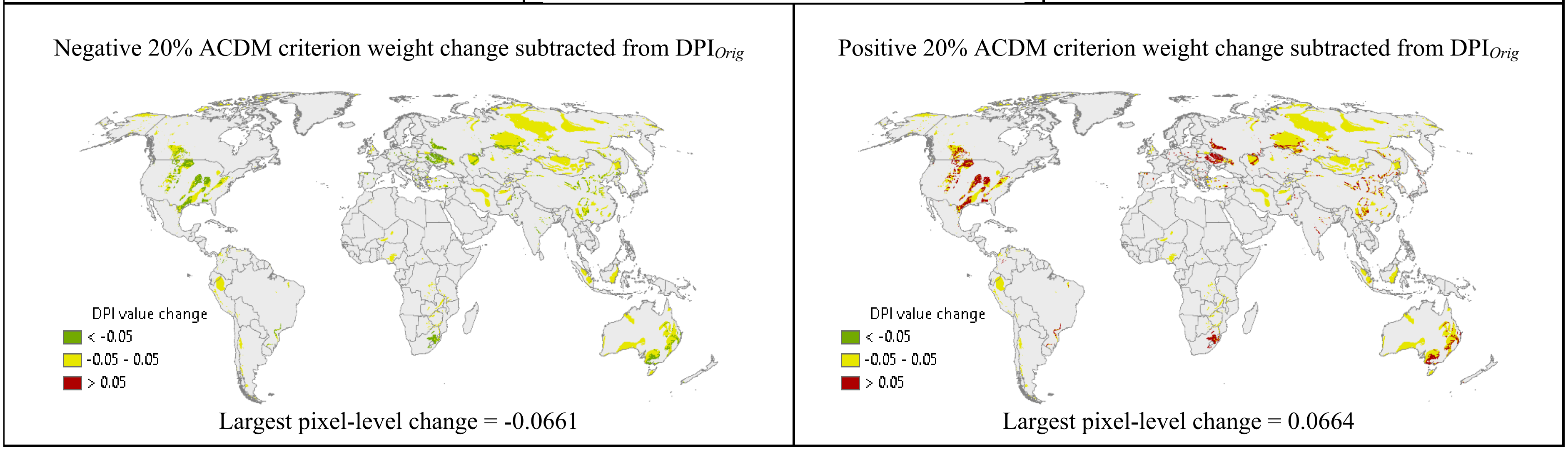

Negative 20% ACDM criterion weight change subtracted from DPI_{Orig}
DPI value change
< -0.05
-0.05 - 0.05
> 0.05
Largest pixel-level change = -0.0661
Positive 20% ACDM criterion weight change subtracted from DPI_{Orig}
DPI value change
< -0.05
-0.05 - 0.05
> 0.05
Largest pixel-level change = 0.0664

References

1. Effat, H. & Effat, H. A. Selection of potential sites for solar energy farms in Ismailia Governorate, Egypt using SRTM and multicriteria analysis. *Int. J. Adv. Remote Sens. GIS* **2**, 205–220 (2013).

2. Janke, J. R. Multicriteria GIS modeling of wind and solar farms in Colorado. *Renew. Energy* **35**, 2228–2234 (2010).

3. Deng, Y. Y. *et al.* Quantifying a realistic, worldwide wind and solar electricity supply. *Glob. Environ. Chang.* **31**, 239–252 (2015).

4. Hernandez, R. R., Hoffacker, M. K., Murphy-Mariscal, M. L., Wu, G. C. & Allen, M. F. Solar energy development impacts on land cover change and protected areas. *Proc. Natl. Acad. Sci. U. S. A.* **112**, 13579–84 (2015).

5. IREA. *Renewable Power Generation Costs in 2014*. (IRENA, 2015).

6. Gorsevski, P. V. *et al.* A group-based spatial decision support system for wind farm site selection in Northwest Ohio. *Energy Policy* **55**, 374–385 (2013).

7. Miller, A. & Li, R. A geospatial approach for prioritizing wind farm development in Northeast Nebraska, USA. *ISPRS Int. J. Geo-Information* **3**, 968–979 (2014).

8. Cotrell, J. *et al. Analysis of Transportation and Logistics Challenges Affecting the Deployment of Larger Wind Turbines: Summary of Results*. (NREL, 2014).

9. Kumar, Y. *et al.* Wind energy: Trends and enabling technologies. *Renew. Sustain. Energy Rev.* **53**, 209–224 (2016).

10. Luo, G., Li, Y., Tang, W. & Wei, X. Wind curtailment of China′s wind power operation: Evolution, causes and solutions. *Renew. Sustain. Energy Rev.* **53**, 1190–1201 (2016).

11. Jager, H. I., Efroymson, R. A., Opperman, J. J. & Kelly, M. R. Spatial design principles for sustainable hydropower development in river basins. *Renew. Sustain. Energy Rev.* **45**, 808–816 (2015).

12. Miao, C., Borthwick, A., Liu, H. & Liu, J. China’s policy on dams at the crossroads: Removal or further construction? *Water* **7**, 2349–2357 (2015).

13. Kucukali, S. Risk assessment of river-type hydropower plants using fuzzy logic approach. *Energy Policy* **39**, 6683–6688 (2011).

14. Paish, O. Small hydro power: technology and current status. *Renew. Sustain. Energy Rev.* **6**, 537–556 (2002).

15. Zarfl, C., Lumsdon, A. E., Berlekamp, J., Tydecks, L. & Tockner, K. A global boom in hydropower dam construction. *Aquat. Sci.* **77**, 161–170 (2014).

16. Strager, M. P. *et al.* Combining a spatial model and demand forecasts to map future surface coal mining in Appalachia. *PLoS One* **10**, e0128813 (2015).

17. Rutledge, D. Estimating long-term world coal production with logit and probit transforms. *Int. J. Coal Geol.* **85**, 23–33 (2011).

18. US Energy Information Administration. *Annual Coal Distribution Report 2016*. (US EIA, 2017).

19. Mutchek, M., Cooney, G., Pickenpaugh, G., Marriott, J. & Skone, T. Understanding the contribution of mining and transportation to the total life cycle impacts of coal exported from the United States. *Energies* **9**, 559 (2016).

20. Bleiwas, D. I. *Estimates of Electricity Requirements for the Recovery of Mineral Commodities, with Examples Applied to Sub-Saharan Africa*. (USGS, 2011).

21. Finer, M. *et al.* Future of oil and gas development in the western Amazon. *Environ. Res. Lett.* **10**, 024003 (2015).

22. Henderson, J. & Loe, J. *The Prospects and Challenges for Arctic Oil Development*. (2014).

23. US Energy Information Administration. *Trends in U.S. Oil and Natural Gas Upstream Costs*. (US EIA, 2016).

24. Petak, K. *et al. U.S. Oil and Gas Infrastructure Investment Through 2035*. (API, 2017).

25. Finer, M., Jenkins, C. N. & Powers, B. Potential of best practice to reduce impacts from oil and gas projects in the amazon. *PLoS One* **8**, e63022 (2013).

26. Ríos-Mercado, R. Z. & Borraz-Sánchez, C. Optimization problems in natural gas transportation systems: A state-of-the-art review. *Appl. Energy* **147**, 536–555 (2015).

27. US Department of Energy. *Impact of Increasing US LNG Exports*. (DOE, 2015).

28. Holditch, S. A. Unconventional oil and gas resource development – Let's do it right. *J. Unconv. Oil Gas Resour.* **1–2**, 2–8 (2013).

29. Schaffartzik, A., Mayer, A., Eisenmenger, N. & Krausmann, F. Global patterns of metal extractivism, 1950–2010: Providing the bones for the industrial society's skeleton. *Ecol. Econ.* **122**, 101–110 (2016).

30. Kogel, J. E. ., Trivedi, N. . & Herpfer, M. A. . Measuring sustainable development in industrial minerals mining. *Int. J. Min. Miner. Eng.* **5**, 4–18 (2014).

31. The Mining Association of Canada. *Levelling the Playing Field Supporting Mineral Exploration and Mining in Remote and Northern Canada*. (The Mining Association of Canada, 2015).

32. Robinson, G. R. & Menzie, W. D. *Economic Filters for Evaluation Porphyry Copper Depoist Respirce Assess,emts Isomg Grade-Tonage Deposit Models, with Examples from the US Geological Survey Global Mineral Resource Assessment*. (USGS, 2014).

33. Choi, Y. & Song, J. Review of photovoltaic and wind power systems utilized in the mining industry. *Renew. Sustain. Energy Rev.* **75**, 1386–1391 (2017).

34. Blachowski, J. Spatial analysis of the mining and transport of rock minerals (aggregates) in the context of regional development. *Environ. Earth Sci.* **71**, 1327–1338 (2014).

35. Karakas, A. & Turner, K. Aggregate supply and demand modeling using GIS methods for the front range urban corridor, Colorado. *Comput. Geosci.* **30**, 579–590 (2004).

36. Robinson, G. R., Kapo, K. E. & Raines, G. L. A GIS analysis to evaluate areas suitable for crushed stone aggregate quarries in New England, USA. *Nat. Resour. Res.* **13**, 143–159 (2004).

37. Robinson, Jr., G. R. & Brown, W. M. *Sociocultural Dimensions of Supply and Demand for Natural Aggregate—Examples from the Mid-Atlantic Region, United States*. (USGS, 2002).

38. Weiss, D. J. *et al.* A global map of travel time to cities to assess inequalities in accessibility in 2015. *Nature* **553**, 333–336 (2018).

39. NOAA National Centers for Environmental Information. Version 4 DMSP-OLS, Stable Nighttime Lights - 2013. http://ngdc.noaa.gov/eog/dmsp/downloadV4composites.html. (2013).

40. Chamberlin, J., Jayne, T. S. & Headey, D. Scarcity amidst abundance? Reassessing the potential for cropland expansion in Africa. *Food Policy* **48**, 51–65 (2014).

41. Lambin, E. F. *et al.* Estimating the world's potentially available cropland using a bottom-up approach. *Glob. Environ. Chang.* **23**, 892–901 (2013).

42. Verburg, P. H., Ellis, E. C. & Letourneau, A. A global assessment of market accessibility and market influence for global environmental change studies. *Environ. Res. Lett.* **6**, 034019 (2011).

43. Villoria, N. B. & Liu, J. Using spatially explicit data to improve our understanding of land supply responses: An application to the cropland effects of global sustainable irrigation in the Americas. *Land use policy* **75**, 411–419 (2018).

44. Lark, T. J., Meghan Salmon, J. & Gibbs, H. K. Cropland expansion outpaces agricultural and biofuel policies in the United States. *Environ. Res. Lett.* **10**, 044003 (2015).

45. Keys, E. & McConnell, W. J. Global change and the intensification of agriculture in the tropics. *Glob. Environ. Chang.* **15**, 320–337 (2005).

46. Saaty, R. W. The analytic hierarchy process—what it is and how it is used. *Math. Model.* **9**, 161–176 (1987).

47. Saaty, T. L. *The Analytic Hierarchy Process*. (McGraw-Hill, 1980).
